# Protective effect of stromal Dickkopf-3 in prostate cancer: opposing roles for TGFBI and ECM-1

**DOI:** 10.1038/s41388-018-0294-0

**Published:** 2018-06-01

**Authors:** Zainab Al Shareef, Hoda Kardooni, Virginia Murillo-Garzón, Giacomo Domenici, Emmanouil Stylianakis, Jennifer H. Steel, Miriam Rabano, Irantzu Gorroño-Etxebarria, Ignacio Zabalza, Maria dM Vivanco, Jonathan Waxman, Robert M. Kypta

**Affiliations:** 1 Department of Surgery and Cancer, Imperial College London, London, UK; 2 Center for Cooperative Research in Biosciences, CIC bioGUNE, Derio, Spain; 3 Department of Pathology, Galdakao-Usansolo Hospital, Galdakao, Spain

## Abstract

Aberrant transforming growth factor–β (TGF-β) signaling is a hallmark of the stromal microenvironment in cancer. Dickkopf-3 (Dkk-3), shown to inhibit TGF-β signaling, is downregulated in prostate cancer and upregulated in the stroma in benign prostatic hyperplasia, but the function of stromal Dkk-3 is unclear. Here we show that *DKK3* silencing in WPMY-1 prostate stromal cells increases TGF-β signaling activity and that stromal cell-conditioned media inhibit prostate cancer cell invasion in a Dkk-3-dependent manner. *DKK3* silencing increased the level of the cell-adhesion regulator TGF-β–induced protein (TGFBI) in stromal and epithelial cell-conditioned media, and recombinant TGFBI increased prostate cancer cell invasion. Reduced expression of Dkk-3 in patient tumors was associated with increased expression of TGFBI. *DKK3* silencing reduced the level of extracellular matrix protein-1 (ECM-1) in prostate stromal cell-conditioned media but increased it in epithelial cell-conditioned media, and recombinant ECM-1 inhibited TGFBI-induced prostate cancer cell invasion. Increased *ECM1* and *DKK3* mRNA expression in prostate tumors was associated with increased relapse-free survival. These observations are consistent with a model in which the loss of Dkk-3 in prostate cancer leads to increased secretion of TGFBI and ECM-1, which have tumor-promoting and tumor-protective roles, respectively. Determining how the balance between the opposing roles of extracellular factors influences prostate carcinogenesis will be key to developing therapies that target the tumor microenvironment.

## Introduction

Signals from cancer cells convert benign stroma to cancer stroma, creating an environment that facilitates tumor progression [1]. However, the tumor microenvironment also contains proteins that can improve patient prognosis [2]. Dickkopf-3 (Dkk-3) is a secreted glycoprotein that is downregulated in prostate cancer [3–6]. Prostate glands of *Dkk3* mutant mice exhibit changes in prostate tissue organization and increased prostate epithelial cell proliferation, suggesting that Dkk-3 is required to maintain a normal microenvironment and that its loss could play a role in cancer progression [4, 7]. In addition, ectopic expression of Dkk-3 inhibits prostate cancer cell proliferation and invasion [4, 7], and an adenoviral vector expressing Dkk-3, Ad-REIC, has shown promise as a therapy for prostate cancer in early stage trials [8, 9]. Dkk-3 is also expressed in prostate stroma, with increased levels reported in benign prostatic hyperplasia (BPH) and prostate cancer [6]. Knockdown of Dkk-3 in primary prostate smooth muscle cells reduces their proliferation and differentiation [10]. However, it is not known if stromal Dkk-3 plays a protective or tumor-promoting role in prostate disease. In addition, Dkk-3 is upregulated in the tumor endothelium, suggesting it plays a role in angiogenesis [11–13].

Knockdown of DKK3 in prostate epithelial cells disrupts their ability to form acini in 3D cultures, and this can be rescued by inhibition of TGF-β/Smad signaling [7]. TGF-β signaling plays an important role in prostate tissue homeostasis [1], and its aberrant activation leads to expression of pro-invasive factors, such as matrix metalloproteases (MMPs) [14]. Notably, Dkk-3 inhibits MMP expression and activity, and MMP inhibitors rescue the effects of DKK3 knockdown on prostate epithelial cell acinar morphogenesis [15]. Based on these studies, we have proposed that endogenous Dkk-3 plays a protective role in prostate cancer by limiting TGF-β/Smad/MMP signaling [16]. However, the loss of Dkk-3 is anticipated to have effects on the activity and/or expression of other proteins in the tumor microenvironment. In this study, we show that the expression level of stromal Dkk-3 is also relevant to prostate cancer, and we identify two secreted proteins, TGFBI (Transforming Growth Factor Beta Induced) and ECM-1 (extracellular matrix protein 1), whose levels are differentially affected by DKK3 silencing in prostate stromal cells and that appear to play opposing roles in prostate cancer.

## Results

### Reduced expression of Dkk-3 in prostate cancer stroma

**Fig. 1:**
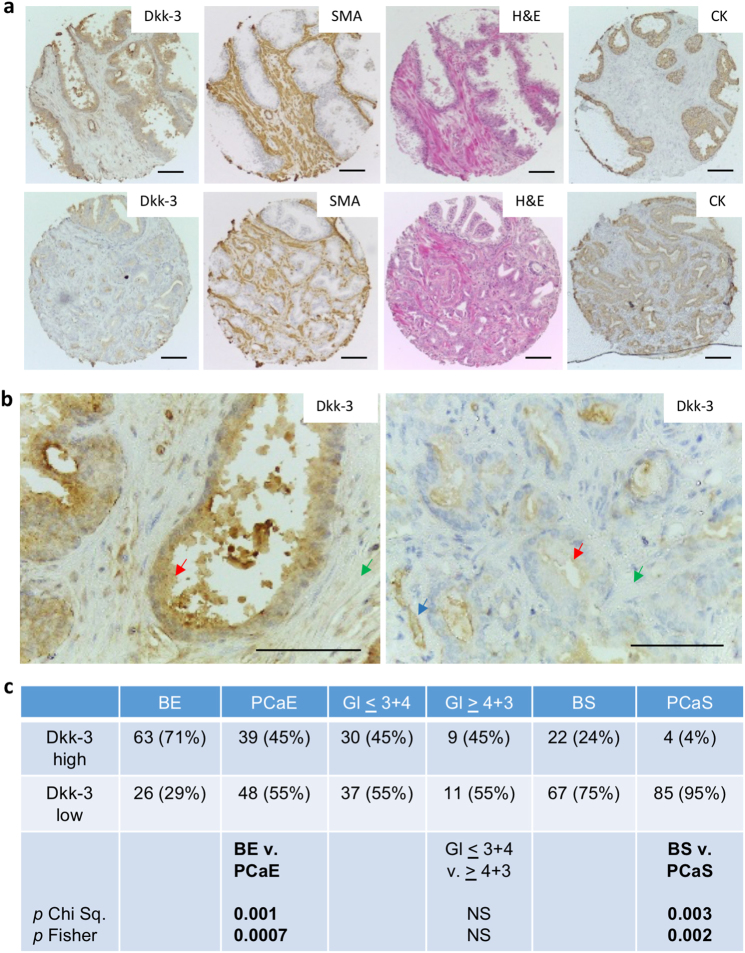
Reduced levels of Dkk-3 in prostate cancer and prostate cancer stroma. **a** Prostate sections from the same patient containing benign (top) or tumor (bottom) tissue were stained for Dkk-3, smooth muscle actin (SMA), hematoxylin and eosin (H&E) and epithelial cytokeratins (CK). **b** Higher magnification images for Dkk-3 staining; arrows indicate Dkk-3 in epithelial (red), stromal (green), and endothelial (blue) cells; scale bars 112 µm. **c** Statistical analysis of Dkk-3 expression; *Gl* Gleason, *PCaE* prostate cancer epithelium, *BS* benign stroma, *PCaS* prostate cancer stroma, *BE* benign epithelium; *p* values from Chi square, Yates correction, and/or Fisher’s Exact test, two-sided

Dkk-3 is abundant in the normal prostate epithelium and downregulated in prostate cancer [3, 4, 6]. Changes in the expression of Dkk-3 have also been reported in benign prostatic hyperplasia [10], but less is known about the expression of Dkk-3 in cancer stroma. We used immunohistochemistry to compare Dkk-3 levels in epithelial and stromal cells in cancer and benign tissue from 99 treatment-naive prostate cancer patients (Supplementary Table 3). Dkk-3 levels in stromal and epithelial cells were scored independently to account for lower expression levels in prostate stroma (Supplementary Figure 1). Near-adjacent sections were stained for smooth muscle actin and vimentin to detect reactive stroma [17] and with pan cytokeratin antibodies to detect epithelial/cancer cells. An example of a patient with moderate Dkk-3 expression in benign epithelium and low Dkk-3 expression in tumor epithelium and tumor stroma, with some expression in endothelial cells is shown in (Fig. 1a, b). Statistical analysis indicated that Dkk-3 expression was not only lower in tumor epithelium than in benign epithelium, but was also lower in tumor stroma than in benign stroma (Fig. 1c).

### DKK3 gene silencing increases of TGF-β signaling in prostate stromal cells

**Fig. 2:**
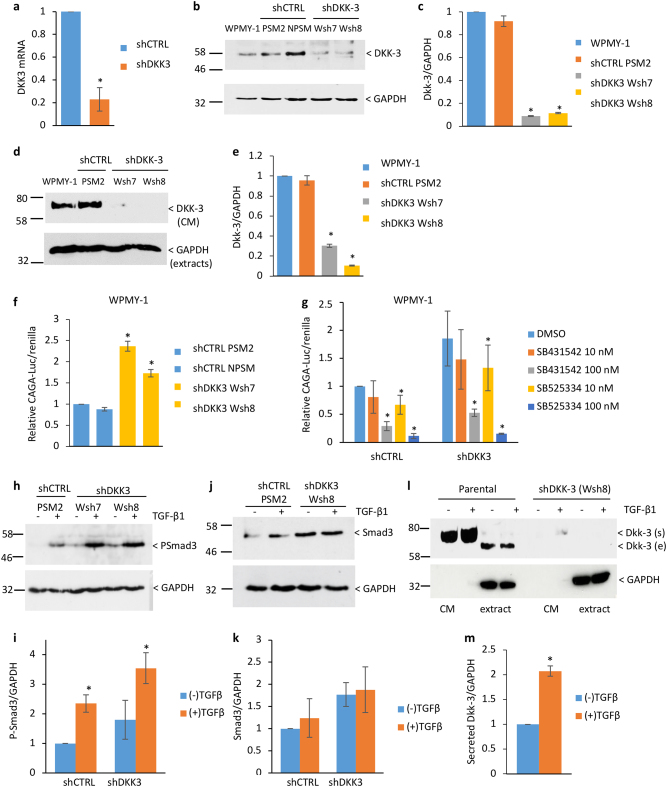
DKK3 gene silencing increases TGF-β signaling in prostate stromal cells. **a** q-RT-PCR analysis showing average relative expression of *DKK3* mRNA in shCTRL (PSM2/PSM3) and shDKK3 (Wsh8/Wsh7) cells; *p < 0.001 by Student’s *t* test. **b** Western blots of extracts from parental, shCTRL (PSM2, NPSM), and shDKK3 (Wsh7 and Wsh8) WPMY-1 cells probed for Dkk-3 and GAPDH; the position of Dkk-3 is indicated. **c** Densitometry analysis of Dkk-3 normalized to GAPDH, error bars show standard deviation (SD), *n* = 7, *p < 0.01 compared to WPMY-1. **d** Western blots of conditioned media (CM) from the indicated cell lines were probed for Dkk-3; the position of secreted Dkk-3 is indicated GAPDH in cell extracts was used as a loading control. **e** Densitometry analysis of Dkk-3 in CM normalized to GAPDH in extracts; error bars represent SD, *n* = 4, *p < 0.0001 compared to WPMY-1. **f** Gene reporter assays for shCTRL (PSM2, NPSM) and shDKK3 (Wsh7, Wsh8) WPMY-1 cells transfected with CAGA-Luc and renilla; error bars show SD, *n* = 3, *p < 0.01, compared to shCTRL PSM2. Graph shows average CAGA-luc/renilla ratios normalized to untreated PSM2 control cells, error bars show SD, *n* = 3, *p < 0.05. **g** Gene reporter assays for shCTRL (PSM3) and shDKK3 (Wsh8) WPMY-1 cells transfected with CAGA-Luc and renilla and treated with vehicle (DMSO) or the indicated concentrations of the TGFBR1 inhibitors SB431542 and SB525334; error bars show SD, *n* = 3 (100 nM), *n* = 6 (10 nM), *p < 0.05 compared to DMSO in each cell line. **h** Western blots of extracts from shCTRL (PSM2) and shDKK3 (Wsh7, Wsh8) WPMY-1 cells cultured for 24 h with (+) or without (−) 10 ng/ml TGF-β1 probed for pSmad3 and GAPDH. **i** Densitometry analysis of pSmad3 normalized to GAPDH; *n* = 4, *p < 0.05. **j** Western blots of extracts from shCTRL (PSM2) and shDKK3 (Wsh8) WPMY-1 cells cultured as in **h** probed for Smad3 and GAPDH. **k** Densitometry analysis of Smad3 normalized to GAPDH; *n* = 4. **l** Western blots of extracts and CM from parental and shDKK3 (Wsh8) WPMY-1 cells as in **h** probed for Dkk-3 and GAPDH. **m** Densitometry analysis of Dkk-3 in CM, normalized to GAPDH in extracts; *n* = 7, *p < 0.05

To investigate the function of stromal Dkk-3, we used the prostate stromal cell line WPMY-1, which is derived from the benign prostate of the same donor as RWPE-1 prostate epithelial cells and has been used as a model to investigate communication between prostate stromal cells and prostate epithelial and prostate cancer cells [18, 19]. WPMY-1 cell lines stably expressing DKK3 shRNA (WPMY-1 shDKK3 Wsh7 and Wsh8) and control shRNA (WPMY-1 shCTRL PSM2, PSM3, NPSM) were generated. Q-PCR confirmed downregulation of *DKK3* mRNA (Fig. 2a). Dkk-3 in cell extracts was approximately 10-fold less abundant in shDKK3 cells than in parental and shCTRL cells (Fig. 2b, c) and 3.5-fold (Wsh7 cells) to 10-fold (Wsh8 cells) less abundant in shDKK3 cell cell-conditioned media (CM) than in parental and shCTRL cell CM (Fig. 2d, e and Supplementary Figure 2a). *DKK3* silencing did not affect WPMY-1 cell proliferation (Supplementary Figure 2b).

Given the effects of Dkk-3 on TGF-β signaling [7] and the importance of TGF-β signaling in stromal cell differentiation [1], we determined the effect of DKK3 silencing on TGF-β signaling in WPMY-1 cells. TGF-β/Smad-dependent gene reporter activity was higher in DKK3-silenced cells than in control cells (Fig. 2f) and was strongly reduced by the TGFBR1 inhibitors SB431542 and SB525334 (Fig. 2g). A significant effect was observed for 10 nM SB525334, consistent with inhibition of TGFBR1/ALK5 (IC50 14 nM), rather than ALK4/7 (IC50 59 nM). In addition, DKK3-silenced cells had higher levels of phosphorylated Smad3 and a trend for higher levels of Smad3 (Fig. 2h–k), whereas Smad2 and Smad4 were not affected (Supplementary Figure 3). In contrast to what has been observed in primary prostate stromal cells [10, 15], TGF-β and DKK3-silencing did not significantly affect MMP2 and SMA expression in WPMY-1 cells, although there was a trend for increased MMP2 levels in CM from DKK3-silenced cells (Supplementary Figure 4). However, TGF-β increased the levels of Dkk-3 in WPMY-1 CM (Fig. 2l, m). In summary, DKK3 silencing increases autocrine TGF-β/Smad3 signaling in WPMY-1 cells but has limited effects on TGF-β induction of MMP2 and SMA, possibly because TGF-β increases Dkk-3 secretion.

### Contrasting effects of DKK3 gene silencing on gene expression in prostate stromal and epithelial cells

**Fig. 3:**
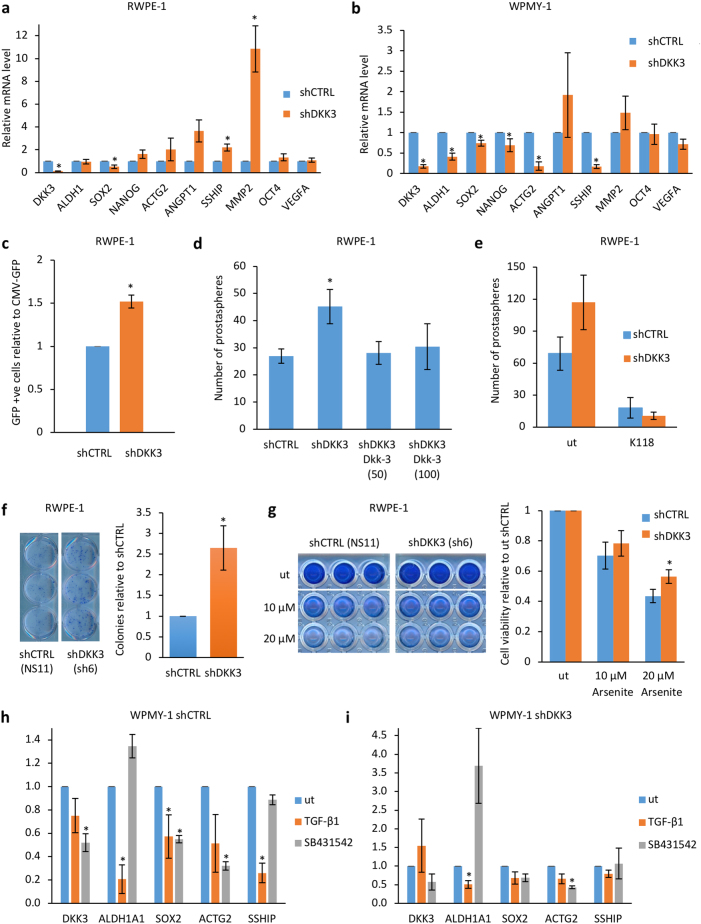
Contrasting effects of DKK3 gene silencing on gene expression in prostate stromal and epithelial cells. **a** q-RT-PCR analysis of the expression of the indicated genes in shCTRL (NS11) and shDKK3 (sh6) RWPE-1 cells, *n* = 3 or 2 (OCT4, VEGF). **b** q-RT-PCR analysis of the expression of the indicated genes in shCTRL (PSM2/3) and shDKK3 (Wsh7/8) WPMY-1 cells; *n* = 3 or 6 (*SOX2*)); error bars show SD, *p < 0.05 compared to shCTRL. **c** Graph showing relative number of GFP-positive cells upon transfection with s-SHIP-GFP, compared to transfection with CMV-GFP, normalized to shCTRL (NS11). **d** Graph showing mean numbers of prostaspheres for shCTRL (NS11) and shDKK3 (sh6) RWPE-1 cells cultured for 7 days, either untreated or in the presence of 50 or 100 ng/ml recombinant Dkk-3; error bars show SD, *p < 0.05, *n* = 3. **e** Graph showing mean numbers of prostaspheres for shCTRL (NS11) and shDKK3 (sh6) RWPE-1 cells cultured for 7 days, either untreated or in the presence of 100 nM SHIP1 inhibitor (K118); error bars show SD, *n* = 2. **f** Left: example of stained colonies from colony formation assays carried out using shCTRL (NS11) and shDKK3 (sh6) RWPE-1 cells plated for 10 days and stained with crystal violet. Graph shows mean colony number, relative to shCTRL, error bars show SD, *n* = 3, *p < 0.05. **g** Cell viability assays using shCTRL (NS11) and shDKK3 (sh6) RWPE-1 cells cultured for 48 h either untreated (ut) or treated with the indicated concentrations of sodium arsenite and stained with crystal violet. Graph shows relative cell number, normalized to ut for each cell line, error bars show SD; *n* = 3, *p* < 0.05 comparing shCTRL and shDKK3 at 20 μM arsenite. **h**, **i** q-RT-PCR analysis of the indicated genes, showing average relative expression in shCTRL (PSM3) (**h**) and shDKK3 (Wsh8) (**i**) WPMY-1 cells either untreated (ut) or treated with 10 ng/ml TGF-β1 or 1 μM SB431542 for 24 h; *n* = 3, *p < 0.05 by Student’s *t* test

Activation of TGF-β signaling in WPMY-1 cells and RWPE-1 cells is accompanied by changes in Smad3 and Smad2 [15], respectively, suggesting there may be cell-type-specific differences in the response to Dkk-3. To study this further, we examined the expression of genes reported to be affected by Dkk-3. These included genes downregulated (*ACTG2*) and upregulated (*ANGPT1*) by DKK3 silencing in stroma [10], upregulated by DKK3 silencing in prostate epithelial cells (*MMP2*) [15] and induced by Dkk-3 in endothelial cells (*VEGFA*) [20]. In addition, since RWPE-1 cell cultures contain a stem/progenitor cell population [21] and Dkk-3 promotes differentiation of embryonic stem cells [22], we examined the stem cell genes *SOX2*, *OCT4 (POU5F1*), and *NANOG*. We also measured the expression of *s-SHIP*, which encodes an isoform of SHIP1 (SH2-containing Inositol 5ʹ-Phosphatase) that lacks the N-terminal domain [23] and is upregulated in RWPE-1 stem/progenitor cells [24] and *ALDH1A1*, a prostate cancer stem cell marker that may be a myofibroblast marker in prostate stroma [25, 26]. In RWPE-1 cells, DKK3 silencing increased *MMP2* expression, as previously observed [15]. It also increased *s-SHIP* expression and showed trends for increasing expression of *ACTG2*, *ANGPT1*, *OCT4*, and *NANOG*, whereas it reduced expression of *SOX2* (Fig. 3a). DKK3 silencing in WPMY-1 cells also reduced *SOX2* expression (Fig. 3b). In addition, there were reductions in *ALDH1A1*, *SOX2*, *NANOG*, *ACTG2*, and *s-SHIP* (Fig. 3b). Thus, DKK3 silencing can have similar (*SOX2*) and opposite (*s-SHIP*) effects on gene expression in prostate stromal and epithelial cells.

To further explore the effect of DKK3 silencing on *s-SHIP*, cells were transfected with GFP plasmids driven by the mouse *s-ship* gene promoter [27], which has been used to select for RWPE-1 stem/progenitor cells [24], or by a control (CMV) promoter. Fluorescence activated cell sorting (FACS) analysis indicated that DKK3 silencing increased the proportion of *s-ship*-GFP-positive cells in RWPE-1 cells (Fig. 3c and Supplementary Figure 5a and b), consistent with increased expression of *s-SHIP* mRNA, while there was no consistent effect in WPMY-1 cells. RWPE-1 cells expressing s-*SHIP* show enhanced sphere-forming capacity and resistance to arsenite-induced cell death [24]. Consistent with this, DKK3 silencing increased RWPE-1 prostasphere formation (Fig. 3d). This increase was blocked by recombinant Dkk-3 **(**Fig. 3d) and by inhibition of SHIP1 (Fig. 3e) at a dose that did not affect RWPE-1 cell viability (Supplementary Figure 5c). In addition, DKK3 silencing increased RWPE-1 cell clonogenicity in colony formation assays (Fig. 3f) and increased resistance of RWPE-1 cells to arsenite-induced cell death (Fig. 3g). Thus, DKK3 silencing in RWPE-1 cells increases prostate epithelial stem/progenitor cell properties that are consistent with the observed increase in the expression of *s-SHIP*.

TGF-β has been reported to inhibit *ALDH1A1* expression in primary prostate stromal cells [25, 26]. To test whether this was also the case in WPMY-1 cells and to test whether other genes affected by DKK3 silencing in WPMY-1 cells are TGF-β-regulated, experiments were carried out in cells treated with TGF-β or SB431542. In control WPMY-1 cells, *ALDH1A1* expression was reduced by TGF-β treatment and showed a trend for increase in SB431542-treated cells (Fig. 3h). TGF-β also reduced *SOX2* and *s-SHIP*. However, SB431542 reduced *SOX2* and *ACTG2* and did not affect *s-SHIP*. SB431542 also reduced *DKK3* expression, which may have effects on expression of genes regulated by DKK3. Examination of these genes in shDKK3 WPMY-1 cells (Fig. 3i) found that *ALDH1A1* expression was further reduced by TGF-β treatment and showed a trend for an increase with SB431542, as seen in control WPMY-1 cells. In contrast, TGF-β did not affect expression of *SOX2* or *s-SHIP*. However, these genes were already expressed at very low levels in shDKK3 WPMY-1 cells. These results suggest that DKK3 silencing has an impact on the expression of TGF-β-repressed genes in WPMY-1 cells.

Taken together, our observations indicate that DKK3 silencing has cell-type-specific effects in RWPE-1 and WPMY-1 cells that reflect alterations in the stem/progenitor cell phenotype of RWPE-1 cells and, in part, on TGF-β signaling in WPMY-1 cells.

### WPMY-1 cell-conditioned media restore normal acinar morphogenesis in a Dkk-3-dependent manner

**Fig. 4:**
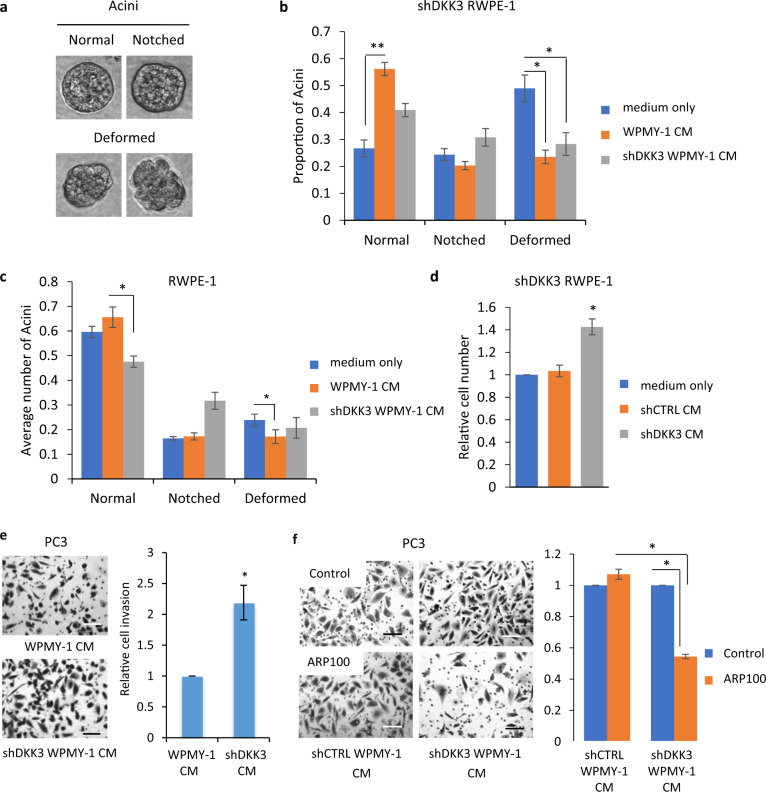
Effects of stromal cell Dkk-3 on acinar morphogenesis and prostate cancer cell invasion. **a** Images of acini used for scoring. **b** Acinar morphogenesis (AM) assays (day 7) using shDKK3 (sh6) RWPE-1 cells cultured with assay medium (control), conditioned media (CM) from WPMY-1 cells or CM from shDKK3 (Wsh8) WPMY-1 cells; error bars show SD, *n* = 4, *p < 0.05, **p < 0.01. Similar results were obtained using shDKK3 NPSM and Wsh7 WPMY-1 cells and using shDKK3 (sh30) RWPE-1 cells (Supplementary Figures 6a and 6b). **c** AM assays (day 7) using shCTRL RWPE-1 (NS11) cells cultured with assay medium (control), CM from WPMY-1 cells or CM from shDKK3 (Wsh8) WPMY-1 cells; error bars show SD, *n* = 3, *p < 0.05; similar results were obtained using shCTRL (NS14) RWPE-1 cells with CM from shCTRL (NPSM) and shDKK3 (Wsh7) WPMY-1 cells (Supplementary Figure 6c). **d** Proliferation assays (48 h) using shDKK3 (sh30) RWPE-1 cells cultured in the upper chamber, separated by a 0.4-μm membrane from the lower chamber, which contained assay media or shCTRL (PSM2) or shDKK3 (Wsh8) WPMY-1 cells; *n* = 4, *p < 0.05. **e** Cell invasion assays using PC3 cells cultured with CM from parental WPMY-1 and shDKK3 (Wsh8) WPMY-1 cells, *n* = 3, *p < 0.01. Left, representative photos of invaded cells stained with crystal violet. Similar results were obtained using CM from shCTRL (NPSM) and shDKK3 (Wsh7) cells (Supplementary Figure 7). **f** Cell invasion assays using PC3 cells cultured with CM from shCTRL (NPSM) and shDKK3 (Wsh8) WPMY-1 cells with or without the MMP2 inhibitor ARP100; *n* = 3, ***p ≤ 0.001. Left: representative photos of invaded cells stained with crystal violet. Scale bars 100 μm

*DKK3* silencing disrupts the ability of prostate epithelial cells to form acini in 3D cultures, an effect that can be rescued by inhibition of TGF-β/Smad signaling [7]. To determine the effects of stromal Dkk-3 on acinar morphogenesis, we treated RWPE-1 cells with conditioned media (CM) collected from control and DKK3-silenced WPMY-1 cells cultured in RWPE-1 cell media for 48 h and carried out acinar morphogenesis assays, scoring for normal, notched, and deformed acini (Fig. 4a). shDKK3 RWPE-1 (sh6) cells mostly formed notched and deformed acini in control medium (Fig. 4b), as previously observed [7]. Addition of parental WPMY-1 cell CM increased the number of normal acini, compared to control, whereas CM from shDKK3 WPMY-1 (Wsh8) cells had no significant effects (Fig. 4b). Similar results were obtained using CM from shCTRL (NPSM) and shDKK3 (Wsh7) WPMY-1 cells (Supplementary Figure 6a) and using a second shDKK3 RWPE-1 cell line (sh30) (Supplementary Figure 6b). Thus, WPMY-1 cell CM restore normal acinar morphogenesis in a Dkk-3-dependent manner.

To determine whether stromal cells also have a general effect on acinar morphogenesis, experiments were repeated using RWPE-1 cells, which express Dkk-3 and form mostly normal acini [7]. Compared to medium alone, WPMY-1 cell CM slightly improved acinar morphogenesis of RWPE-1 cells, whereas shDKK3 WPMY-1 cell CM reduced it (Fig. 4c). Similar results were obtained when comparing shCTRL (NPSM) and shDKK3 (Wsh7) WPMY-1 cell CM treatment of shCTRL (NS14) RWPE-1 cells (Supplementary Figure 6c). These results suggest that shDKK3 WPMY-1 cell CM contains a factor that disrupts acinar morphogenesis. Acinar morphogenesis can be disrupted by EGF-induced RWPE-1 cell proliferation [28]. We therefore carried out co-culture assays to determine whether WPMY-1 cells secrete factors affecting RWPE-1 cell proliferation. Compared with medium alone, co-culture with shCTRL WPMY-1 cells did not affect RWPE-1 cell proliferation. In contrast, co-culture with shDKK3 WPMY-1 cells increased RWPE-1 cell proliferation (Fig. 4d). These results suggest that DKK3-silenced WPMY-1 cells secrete a factor that increases RWPE-1 cell proliferation that might account for the disruption of acinar morphogenesis by DKK3-silenced WPMY-1 cell CM.

### DKK3-silenced WPMY-1 cell CM increases prostate cancer cell invasion

Dkk-3 inhibits the invasion of PC3 prostate cancer cells [7]. In order to determine the effects of stromal Dkk-3 on cell invasion, PC3 cells were cultured in the presence of CM from control and DKK3-silenced WPMY-1 cells. PC3 cell invasion was significantly higher in the presence of CM from shDKK3 (Wsh8) WPMY-1 cells, compared to CM from parental WPMY-1 cells (Fig. 4e). Similar results were observed when comparing CM from shDKK3 (Wsh7) and shCTRL (NPSM) WPMY-1 cell CM (Supplementary Figure 7a). WPMY-1 cell CM did not significantly affect PC3 cell invasion, compared to medium alone (Supplementary Figure 7b). These results are consistent with a model in which the loss of Dkk-3 in stromal cells leads to the activation or secretion of factors that promote prostate cancer cell invasion. Given the trend for increased MMP2 in shDKK3 WPMY-1 cell CM (Supplementary Figure 4a and b), we tested the effects of the MMP2 inhibitor ARP100. ARP100 did not affect invasion in the presence of WPMY-1 CM but it reduced PC3 cell invasion in the presence of shDKK3 WPMY-1 cell CM (Fig. 4f), consistent with MMP2 activity playing a role in the increase in invasion observed in the presence of DKK3-silenced WPMY-1 cell CM.

### Identification of TGFBI and ECM-1 as secreted proteins affected by DKK3-silencing in WPMY-1 and RWPE-1 cells

**Fig. 5:**
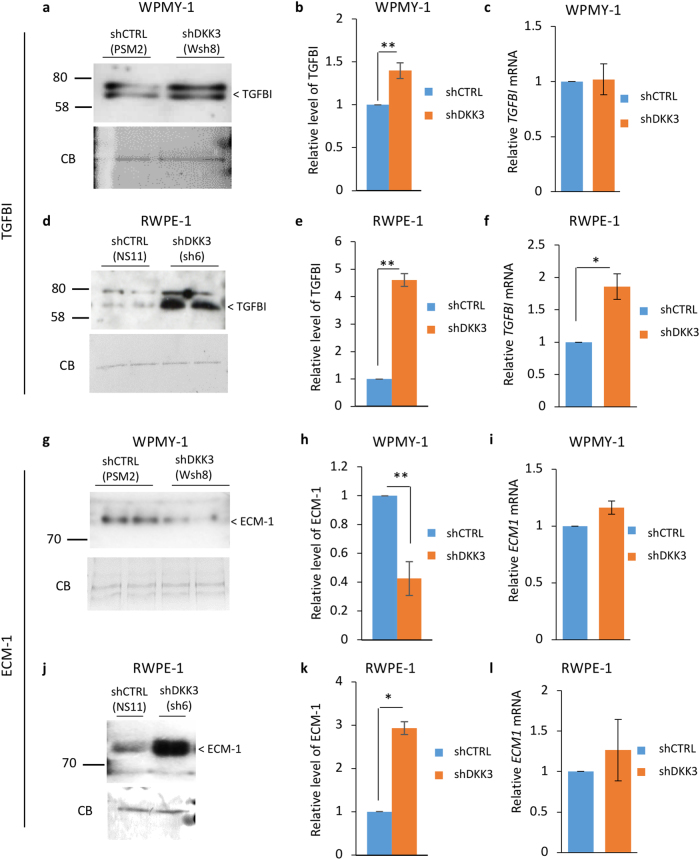
Effects of DKK3 silencing on TGFBI and ECM-1 protein and mRNA levels in WPMY-1 and RWPE-1 cells. **a** Western blots of CM from equal numbers of shCTRL (PSM2) and shDKK3 (Wsh8) WPMY-1 cells cultured in serum-free medium for 48 h were probed for TGFBI; a Coomassie Blue (CB)-stained gel of samples run in parallel was used as a loading control. **b** Densitometry analysis of TGFBI in CM; graph shows average intensity normalized to PSM2, error bars show SD, **p < 0.01, *n* = 5. **c** q-RT-PCR analysis of *TGFBI* mRNA levels, relative to *36B4*, in shCTRL (PSM2/PSM3) and shDKK3 (Wsh7/Wsh8) WPMY-1 cells; error bars show SD. **d** Western blots of CM from equal numbers of shCTRL (NS11) and shDKK3 (sh6) RWPE-1 cells cultured in serum-free medium for 48 h were probed for TGFBI; a CB-stained gel of samples run in parallel was used as a loading control. **e** Densitometry analysis of TGFBI in CM; graph shows average intensity normalized to NS11, error bars show SD, **p < 0.01, *n* = 3. **f** q-RT-PCR analysis of *TGFBI* mRNA levels, relative to *36B4*, in shCTRL (NS11) and shDKK3 (sh6) RWPE-1 cells; error bars show SD, *n* = 3, *p < 0.05. **g**–**l** ECM-1 protein and *ECM1* mRNA levels were analyzed as in **a**–**f**; **h** **p < 0.01, *n* = 6, **k** *p < 0.05, *n* = 3

To identify proteins involved in the response to Dkk-3, CM from control and DKK3-silenced WPMY-1 and RWPE-1 cells were compared using an antibody array (Supplementary Figure 8). Two proteins affected by *DKK3* silencing, TGFBI and ECM1, were chosen for further study. Western blotting and q-PCR were carried out to validate the antibody array results. TGFBI was detected as a doublet at 68-70 kDa (Fig. 5a), the reported size of TGFBI, and was more abundant in CM from DKK3-silenced WPMY-1 cells than control cells (Fig. 5b). DKK3 silencing did not affect *TGFBI* mRNA levels in WPMY-1 cells (Fig. 5c). There were also higher levels of TGFBI in CM from DKK3-silenced RWPE-1 cell than in control cell CM (Fig. 5d, e). In contrast to WPMY-1 cells, *TGFBI* mRNA levels were higher in DKK3-silenced cells than in control RWPE-1 cells (Fig. 5f). ECM-1 was detected as a protein of 85 kDa (Fig. 5g), the reported size of ECM-1, and was lower in DKK3-silenced WPMY-1 cell CM than in control cell CM (Fig. 5h). DKK3 silencing did not affect *ECM1* mRNA levels (Fig. 5i). In contrast to WPMY-1 cells, ECM-1 levels were higher in DKK3-silenced RWPE-1 cell CM than in control RWPE-1 CM (Fig. 5j, k). Again, there was no effect on *ECM1* mRNA levels (Fig. 5l). In summary, *DKK3* silencing increased TGFBI levels in both WPMY-1 and RWPE-1 cells CM, whereas it had opposite effects on the levels of ECM-1, increasing it in RWPE-1 cell CM and reducing it in WPMY-1 cell CM.

### TGFBI and ECM-1 have opposite effects on acinar morphogenesis and PC3 cell invasion

**Fig. 6:**
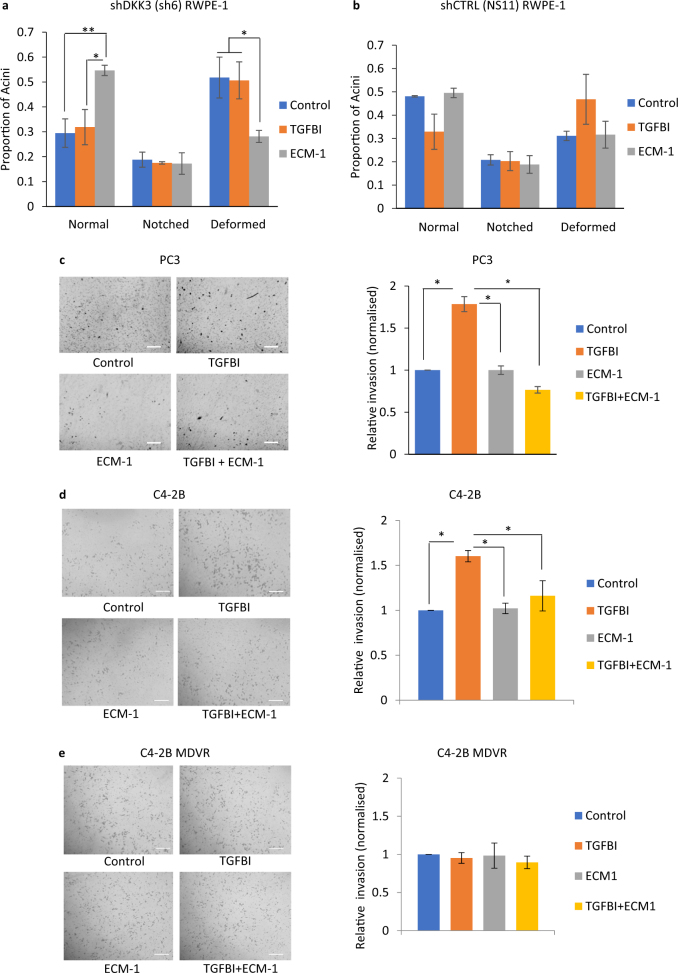
TGFBI and ECM-1 have opposite effects on acinar morphogenesis and PC3 cell invasion. **a** Acinar morphogenesis assays for shDKK3 (sh6) RWPE-1 cells cultured in media only (control) or with ECM-1 (100 ng/ml) or TGFBI (1 μg/ml) for 7 days; error bars show SD, *p < 0.05, **p < 0.01, *n* = 4. **b** Acinar morphogenesis assays for shCTRL (NS11) RWPE-1 cells cultured as in **a**. **c** Invasion assays for PC3 cells plated in triplicate wells for 24 h in serum-free RPMI with PBS (Control) or with TGFBI (1 μg/ml), ECM-1 (100 ng/ml) or both TGFBI and ECM-1. Left, example photos of invaded cells, right, graph showing average number of invaded cells, normalized to control, error bars show SD, *n* = 3, **p < 0.01 by ANOVA; scale bars 100 μm. **d**, **e** Invasion assays for C4-2B cells (**d**) and enzalutamide-resistant C4-2B (MDVR) cells (**e**) treated as in **c**

In order to determine the possible functions of TGFBI and ECM-1, we examined their effects on acinar morphogenesis. Cells were plated for acinar morphogenesis assays and treated with media containing recombinant purified TGFBI or ECM-1 at concentrations that we had determined to be similar to those found in cell CM. Acinar morphogenesis of DKK3-silenced RWPE-1 cells was not affected by addition of TGFBI but was significantly improved by addition of ECM-1, which increased the number of normal acini and reduced the number of deformed acini (Fig. 6a). In contrast, ECM-1 did not affect acinar morphogenesis of control RWPE-1 cells, whereas addition of TGFBI showed trends for reducing the number of normal acini and increasing the number of deformed acini (Fig. 6b).

Since DKK3-silenced WPMY-1 cell CM increased PC3 cell invasion and contained more TGFBI and less ECM-1 than control cell CM, we hypothesized that exogenous TGFBI and/or ECM-1 would affect PC3 cell invasion. PC3 cell invasion assays were therefore carried out in control medium or media containing recombinant TGFBI, ECM-1, or both proteins together (Fig. 6c). Compared to control, TGFBI significantly increased PC3 cell invasion, while ECM-1 had no effect. However, ECM-1 inhibited TGFBI-promoted cell invasion. There were no effects of either protein on cell proliferation (results not shown). To determine if these effects could be observed in another metastatic prostate cancer cell line, experiments were carried out using C4-2B cells, a metastatic androgen-insensitive derivative of LNCaP [29]. Similar to PC3 cells, C4-2B cell invasion was increased by TGFBI treatment and this response was inhibited by ECM-1 (Fig. 6d). Given the recent report linking androgen signaling to TGFBI [30], experiments were also carried out using enzalutamide-resistant C4-2B cells (C4-2B MDVR). In contrast to PC3 and C4-2B, C4-2B MDVR cell invasion was not significantly affected by TGFBI or ECM-1 (Fig. 6e). In summary, in the contexts of prostate epithelial cell acinar morphogenesis and prostate cancer cell invasion, TGFBI and ECM-1 have tumor-promoting and tumor-suppressing functions, respectively. The improvement in RWPE-1 acinar morphogenesis and the inhibition of TGFBI-induced PC3 and C4-2B cell invasion by exogenous ECM-1 suggests that increased expression of ECM-1 in prostate cancer may be beneficial to patients, whereas the opposite may be true for TGFBI. The lack of a significant effect of either TGFBI or ECM-1 on invasion of enzalutamide-resistant C4-2B cells suggests that the cellular response to these proteins may be altered in treatment-resistant cells.

### Correlations among the expression of Dkk-3, TGFBI, and ECM-1 in patient tumors

**Fig. 7:**
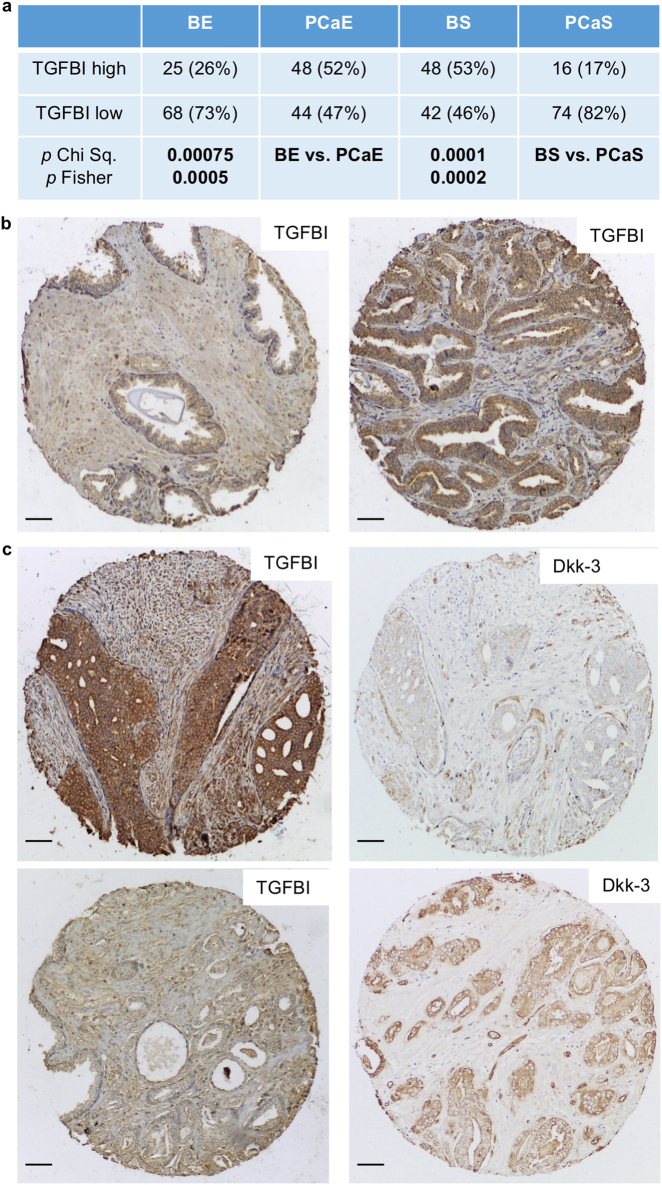

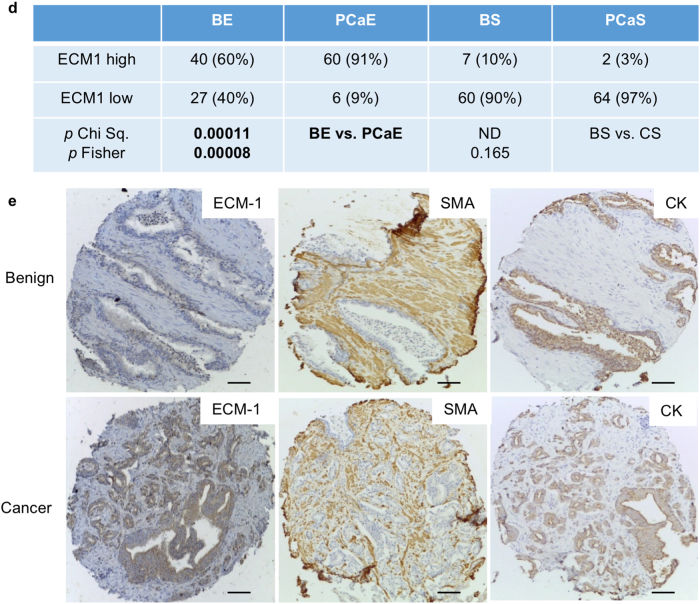
Analysis of TGFBI and ECM-1 in prostate cancer patients. **a** Statistical analysis of TGFBI expression in benign prostate and prostate cancer; **b** Benign and tumor sections from a patient stained for TGFBI. **c** Tumor sections from two patients stained for TGFBI and Dkk-3; scale bars 65 µm. **d** Statistical analysis of ECM-1 expression in benign prostate and prostate cancer. **e** Example of a patient tumor with increased ECM-1 in cancer, compared to in benign epithelium. Benign and cancer sections from the same patient were stained for ECM-1, SMA, and pan-CK; scale bars 84 µm. *Gl* Gleason, *PCaE* prostate cancer epithelium, *BS* benign stroma, *PCaS* prostate cancer stroma, *BE* benign epithelium; *Chi Sq.* Chi square Yates correction, Fisher, Fisher’s Exact test, two-sided

In order to determine whether links between Dkk-3, TGFBI, and ECM-1 could also be found in patient tumors, we compared their expression levels by immunohistochemistry. The scoring system used is shown in Supplementary Figures 9 and 10. TGFBI was more prevalent in cancer than in benign epithelium and was found at lower levels in tumor stroma than in benign stroma (Fig. 7a, b). Comparison of TGFBI and Dkk-3 staining in cancer found high levels of TGFBI in some tumors that expressed low levels of Dkk-3 (Fig. 7c), suggesting an inverse correlation. Statistical analysis confirmed this in low Gleason score tumors but not in cancer stroma or benign tissue (Table 1). ECM-1 staining was also higher in tumor epithelium than in benign epithelium (Fig. 7d, e). There was no correlation between Dkk-3 and ECM-1 in cancer, although there were correlations in tumor stroma (Table 1).

**Table 1:** Correlation of Dkk-3 with TGFBI and ECM-1 in patient tumors

Comparison	Correlation coefficient	*p*
Dkk-3 PCaE vs. TGFBI PCaE	**−0.305**	**0.0037**
Gl. ≥ 43	−0.1794	0.437
Gl. ≤ 34	**−0.2986**	**0.014**
Dkk-3 PCaCS vs. TGFBI PCaCS	0.2028	0.057
Gl. ≥ 43	−0.1077	0.642
Gl. ≤ 34	0.0464	0.709
Dkk-3 BE vs. TGFBI BE	−0.0638	0.553
Dkk-3 BS vs. TGFBI BS	0.1792	0.093
ECM1 PCaE vs. Dkk-3 PCaE	0.069	0.58
ECM1 PCaS vs. Dkk-3 PCaS	**0.396**	**0.001**
Gl. ≥ 43	0.177	0.48
Gl. ≤ 34	**0.514**	**0.0002**
ECM-1 BS vs. Dkk-3 BS	0.009	0.942
ECM-1 BE vs. DKK3 BE	0.133	0.308
ECM-1 PCaE vs. TGFBI PCaE	0.239	0.052
ECM-1 PCaS vs. TGFBI PCaS	**0.367**	**0.002**
Gl. ≥ 43	0.433	0.072
Gl. ≤ 34	**0.3445**	**0.018**
ECM1 BE vs TGFBI BE	−0.152	0.241
ECM1 BS vs. TGFBI BS	0.126	0.331

Numbers in bold highlight statistically significant differences

To determine whether *DKK3*, *TGFBI*, and *ECM1* mRNA expression is associated with prostate cancer patient survival, we used PROGgene (http://www.compbio.iupui.edu/proggene) [31, 32] and the Prostate cancer dataset GSE70768 [33]. Correlation of gene expression with respect to relapse-free survival of prostate cancer patients (*n* = 110) was analyzed by taking the median gene expression value for each gene and then dividing patients into high (above the median) and low (below the median). Relapse-free survival was higher in patients with high *DKK3* expression than in patients with low *DKK3* expression (Supplementary Figure 11a). High expression of *ECM1* showed a trend for association with relapse-free survival (*p* = 0.17, not shown), and this was significant in a second dataset (GSE70769; *n* = 91) (Supplementary Figure 11b). There were no significant differences in patient survival with respect to *TGFBI* expression (Supplementary Figure 11c). These results are consistent with Dkk-3 and ECM-1 playing a protective role in prostate cancer.

## Discussion

Several studies have reported downregulation of Dkk-3 in prostate cancer [5]. Some of these also noted expression of Dkk-3 in prostate cancer stroma [3, 6], but the relevance of stromal Dkk-3 to prostate cancer was not examined. Given the tumor-inhibitory properties of Dkk-3, we hypothesized that the stromal expression of Dkk-3 is protective and may prevent prostate cancer initiation or progression. Consistent with this, examination of Dkk-3 expression in prostate stroma in benign and tumor sections revealed a significant reduction in the levels of Dkk-3 in tumor stroma, compared to that in stroma of benign tissue. To study the function of stromal Dkk-3, we used WPMY-1 prostate stromal cells as a model system. WPMY-1 cells are derived from benign prostate stroma and their expression profile indicates that they are myofibroblasts [34], the major cell type of reactive stroma in well-differentiated foci of prostate cancer [1]. WPMY-1 cells secreted a high level of Dkk-3 that was further increased by TGF-β treatment. This suggests that there may be a negative feedback loop in which downregulation of Dkk-3 activates TGF-β/Smad signaling, leading to auto-induction of TGF-β1 [35], increased Dkk-3 secretion and subsequent inhibition of TGF-β signaling.

DKK3 silencing increased TGFβ/Smad-dependent transcription both in WPMY-1 and RWPE-1 cells but there were differences. DKK3 silencing increased Smad3 phosphorylation in WPMY-1 cells, whereas it increases Smad2 phosphorylation in RWPE-1 cells. Smad3 also plays a role in the response to Dkk-3 in RWPE-1 cells, however, as the Smad3 inhibitor SIS3 rescues acinar morphogenesis in DKK3-silenced RWPE-1 cells [7]. Common targets of Dkk-3 in WPMY-1 and RWPE-1 cells include TGFBI and possibly MMP2. *TGFBI* and *MMP2* are both TGF-β target genes that are upregulated by *DKK3* silencing in RWPE-1 cells [10]. In WPMY-1 cells, *TGFBI* expression is also increased by TGF-β and reduced by SB431542 treatment (Supplementary Figure 12c), and these responses are blunted in shDKK3 WMPY-1 cells (Supplementary Figure 12d). DKK3 silencing did not appear to affect *TGFBI* or *MMP2* gene expression in WPMY-1 cells, although it increased TGFBI protein levels and showed a trend for increasing MMP2 protein levels. Both TGFBI and MMP2 are highly expressed in WPMY-1 cells, which may make it difficult to observe increases at the mRNA level. Alternatively, DKK3 silencing may increase MMP2 activity, rather than expression. The relevance of increased MMP2 activity for prostate cancer cell invasion is underlined by the ability of the MMP2 inhibitor ARP100 to reduce the pro-invasive effects of DKK3-silenced WPMY-1 CM in PC3 cells. Dkk-3 is itself a proteolytic target of MMP2 [36], providing further possibilities for functional interaction. The function of TGFBI in prostate stromal cells remains to be determined. Treatment of WPMY-1 cells with recombinant TGFBI for 24 h had minimal effects on the expression of genes altered by DKK3 silencing (Supplementary Figures 12a and b), although it did reduce *ALDH1A1* expression in shCTRL WPMY-1 cells. The reduction (29%) was weaker than those of TGF-β1 (79%) or DKK3 silencing (91%), suggesting that TGFBI alone is not responsible for the reduced expression of ALDH1A1 in DKK3-silenced cells.

In addition to these common effects of *DKK3* silencing in RWPE-1 and WPMY-1 cells, we observed opposite effects on *s-SHIP* expression, suggesting that Dkk-3 plays cell-type-specific roles that relate to cell differentiation. *DKK3* silencing also had opposite effects on ECM-1 protein levels. These differential effects of DKK3 silencing may reflect cell type-specific differences in TGF-β signaling, differential glycosylation or proteolytic processing of Dkk-3 [37, 38], or the expression of cell-type-specific Dkk-3 receptors. Mammalian receptors for Dkk-3 have yet to be identified, but zebrafish Dkk3a, which is 42% identical to human Dkk-3, binds an α6 integrin [39, 40]. Although the results of our experiments using WPMY-1 cell CM are more consistent with Dkk-3 acting extracellularly or via cell-surface receptors, some studies suggest Dkk-3 also acts intracellularly [5]. Recently, an alternative start site in the mouse *Dkk3* promoter was identified that produces an intracellular form of Dkk-3 (Dkk3b) that inhibits cell proliferation by binding to β-catenin in a complex with β-TrCP [41]. However, it is not known if Dkk3b exists in human cells.

TGFBI protein levels were significantly higher in prostate cancer than in benign prostate. There was an inverse correlation of TGFBI and Dkk-3 expression in prostate cancer, consistent with the increased levels of TGFBI observed upon DKK3 silencing in RWPE-1 cells. In contrast, TGFBI expression was lower in tumor stroma, compared to that in benign stroma and did not show an inverse correlation with Dkk-3, in fact there was a trend for a positive correlation. The inverse correlation between TGFBI and Dkk-3 in cancer was only observed in low Gleason score tumors, suggesting that regulation of TGFBI by Dkk-3 is less important in more advanced prostate cancer. This would be consistent with the correlation of high *DKK3* but not low *TGFBI* expression with relapse-free survival in patients. A comparison of TGFBI and Dkk-3 by immunohistochemistry in a larger cohort of patients will be required to determine if this is the case. TGFBI binds to type I, II, and IV collagens and integrins to regulate cell adhesion and migration [42]. Expression of TGFBI in prostate cancer is repressed by promoter methylation [43] and by miR-675, which inhibits prostate cancer migration [44]. In addition, *TGFBI* expression is repressed by the androgen-regulated transcription factor SPDEF [30]. Androgen deprivation was found to increase TGFBI levels and TGFBI knockdown suppressed prostate cancer cell migration and inhibited tumor growth and metastasis [30]. We found that TGFBI increased the invasion of PC3 and C4-2B cells, but not of enzalutamide-resistant C4-2B MDVR cells. The reason for this is not known but it could reflect changes in the expression of TGFBI receptors. We noted that C4-2B cells had a higher capacity for invasion than C4-2B MDVR cells (VMG, unpublished results). Moreover, endogenous *TGFBI* mRNA expression was similarly low in C4-2B and C4-2B MDVR cells (Supplementary Figures 12e and f), suggesting that TGFBI does not play a role in enzalutamide resistance in this cell model.

ECM-1 was the other major protein in CM affected by *DKK3* silencing. *DKK3* silencing had no effect on *ECM1* mRNA levels, suggesting that Dkk-3 affects ECM-1 protein stability or secretion. ECM-1 was difficult to detect in RWPE-1 cell extracts, while in WPMY-1 cell extracts it was not significantly affected by DKK3 silencing, suggesting that most ECM-1 is secreted and that the increase of ECM-1 in CM is a result of increased secretion or stability. ECM-1 associates with several extracellular proteins, including MMP9, which it inhibits [45], and perlecan. ECM-1 and perlecan form a network of basement membrane proteins that also contains collagen IV and laminin [45, 46]. ECM-1 is overexpressed in many types of cancer [47] and in most cases it has a tumor-promoting effect, correlating with increased metastasis and poor prognosis. However, in hepatocellular cancer, ECM1 gene silencing increases anchorage-independent growth [48]. We found that ECM-1 was more highly expressed in prostate cancer than in benign prostate epithelium. However, there was a positive correlation of Dkk-3 and ECM-1 in cancer stroma, and *ECM1* gene expression correlated with increased relapse-free survival of prostate cancer patients, suggesting ECM-1 has a tumor-inhibitory function. The correlation of Dkk-3 and ECM-1 in cancer stroma might reflect a situation where the loss of Dkk-3 in tumor cells leads to increased expression of stromal Dkk-3 and ECM-1 in a homeostatic response that prevents tumor progression. Consistent with this, ECM-1 increased normal acinar morphogenesis in RWPE-1 cells and inhibited the pro-invasive activity of TGFBI in PC3 and C4-2B cells. It is not clear how ECM-1 inhibits TGFBI-induced invasion, but it may inhibit MMP activity [45] or compete with TGFBI for integrin binding. TGFBI binds to integrins via an RGD motif and ECM-1 can compete with RGD peptides to bind αv integrins and block activation of latent TGF-β [49]. PC3 cells express integrin αvβ6, which plays a role in their migration [50], so it is possible that ECM-1 inhibits TGFBI-induced invasion by blocking TGFBI-mediated integrin activation.

In conclusion, our results are consistent with a negative feedback model that implicates Dkk-3 and TGF-β signaling in the regulation of epithelial–stromal interactions taking place during prostate cancer initiation and progression. In this model, expression of Dkk-3 in the benign prostate epithelium prevents aberrant activation of TGF-β signaling. The epigenetic loss of *DKK3* expression activates TGF-β signaling, leading to increased expression of pro-invasive factors, such as TGFBI and MMP2, which have the potential to promote progression to cancer. However, activation of TGF-β signaling also leads to increased secretion of stromal Dkk-3, which provides a first line of defense. The results of our WPMY-1 cell experiments indicate that stromal Dkk-3 can attenuate prostate epithelial cell proliferation, restore normal prostate epithelial architecture (as reflected by the acinar morphogenesis assays) and inhibit prostate cancer cell invasion. The loss of stromal Dkk-3 is therefore predicted to lead to further disruption of prostate architecture, increased proliferation and prostate cancer cell invasion. The correlation of ECM-1 and Dkk-3 expression in WPMY-1 cells, ECM-1 inhibition of TGFBI-dependent prostate cancer cell invasion and the correlation of *ECM1* and *DKK3* expression with relapse-free survival, suggest that ECM-1 also participates in the Dkk-3 defense mechanism.

## Materials and methods

### Cell culture

WPMY-1 cells [34] (ATCC) were cultured in DMEM (Life Technologies) with 10% FCS (First Link Ltd.) and antibiotics (100 units/ml penicillin and 100 μg/ml streptomycin, Sigma). RWPE-1 (ATCC) and RWPE-1 shRNA cell lines (NS11, NS14 expressing control shRNA, sh6 and sh30 expressing DKK3 shRNA) [7] were cultured in keratinocyte serum-free medium (KSFM) supplemented with bovine pituitary extract (BPE) and EGF (Thermo Fisher) and antibiotics, with 0.75 μg/ml puromycin (Sigma) added to shRNA-expressing cells. PC3 cells (ATCC) and C4-2B cells (from Charlotte Bevan, Imperial College London), were cultured in RPMI-1640 with Glutamax (Life Technologies), 10% FCS and antibiotics and were authenticated by DNA profiling (Eurofins Genomics, Germany). All cells were cultured at 37 °C and 5% CO_2_ and tested for mycoplasma (Mycoplasma Detection Kit, Lonza) every 3 months. WPMY-1 cells were transfected with pSM2-based shRNAmir plasmids [7] using Lipofectamine 2000 and selected in medium containing 1.5 μg/ml puromycin. DKK3 shRNA cell lines were derived from single colonies and control shRNA cells from pools of 20-50 colonies. Two DKK3 shRNA clones (shDKK3 Wsh7 and Wsh8) and four control shRNA lines (shCTRL PSM2, PSM3, NPSM) were used for these studies. Enzalutamide-resistant C4-2B cells (C4-2B MDVR) were generated by culturing C4-2B cells in the presence of 20 μM enzalutamide (MDV3100) (Selleckchem) for 2 weeks and then 10 μM MDV3100 for 3 months, after which they were maintained in the presence of 10 μM MDV3100. Control cells were cultured in parallel in the presence of an equal volume of carrier (DMSO).

### Western blotting

Cells (2–4 × 10^5^) were plated in duplicate wells of six-well plates for 48 h prior to preparation of cell extracts. In some experiments, media were changed to serum-free with or without 10 ng/ml TGF-β (R&D Systems) for 24 h. CM were centrifuged at 500 × *g* for 5 min and supernatants added to an equal volume of 2× Laemmli buffer (Sigma). Cells were lysed on ice in RIPA lysis buffer (0.5% Na deoxycholate, 1% Triton X-100, 20 mM Tris pH 8.0, 0.1% SDS, 100 mM NaCl, 50 mM NaF, 1 mM EDTA), cOmplete™ EDTA-free Protease Inhibitor Cocktail (Sigma) and PhosSTOP Phosphatase Inhibitor Cocktail (Sigma)). Lysates were incubated for 15 min on ice, centrifuged at 15,000×*g* for 15 min at 4 °C and added to an equal volume of 2× Laemmli buffer. To determine the levels of TGFBI and ECM-1 in CM, 10^6^ cells per well were plated in six-well plates overnight and the media changed to 1 ml of the appropriate serum-free media (KSFM or DMEM, respectively) for 48 h, prior to collection of CM. Samples were heated on a shaker for 3 min at 95 °C prior to loading on 7.5 or 10% SDS polyacrylamide gels. Transfer of proteins to PVDF membranes was carried out using a semi-dry transfer apparatus (Bio-Rad). Membranes were rinsed in TBST (Tris-buffered saline with 0.1% Tween 20) and incubated in blocking buffer (5% BSA in TBST) at room temperature for 1 h. Primary antibodies (Supplementary Table 1) diluted in blocking buffer were added and membranes were incubated on a rocker in a cold room overnight. Membranes were washed at least three times for 5 min in TBST, incubated with HRP-conjugated donkey secondary antibodies (1:10,000, Stratech Scientific Ltd) in blocking buffer for 1 h, washed three times for 15 min and incubated with Clarity^TM^ ECL solution (Bio-Rad) for 5 min, exposed to X-ray film and developed using an OPTIMAX film processor.

### RNA extraction, cDNA synthesis, and q-RT-PCR

Total RNA was extracted using PureLink RNA Mini Kit (Life technologies). cDNA was synthesized from total RNA using Quantitect Reverse Transcription Kit (Qiagen) according to manufacturer’s instructions. Gene expression was determined by q-PCR using SYBR Green PCR Master Mix (Bio-Rad) and a 7900HT Fast Real-Time PCR thermal cycler (Applied Biosystems) as previously described [15]. The expression levels of target gene were normalized to an endogenous reference gene (*36B4*) and the fold change, as a measure of relative expression, was calculated using the comparative CT (2^–ΔΔ^CT) method [51]. The sequences of the primers used are in Supplementary Table 2.

### Fluorescence activated cell sorting

Cells were plated overnight in six-well plates (10^5^ cells per well) prior to transfection with plasmids encoding GFP driven by a constitutive promoter (CMV) or the mouse s-ship gene promoter (s-SHIP-GFP) [27] using Lipofectamine Plus. After 24 h, transfected cells were washed in PBS, trypsinized, centrifuged in FACS tubes at 500×*g* for 5 min and resuspended in 500 μl 1% BSA in PBS with 7-Amino-Actinomycin D (7-AAD) (Invitrogen) to identify non-viable cells. FACS analysis was carried out using a FACS Aria flow cytometer (Becton Dickinson) and FACS Diva™ Software.

### Acinar morphogenesis, colony formation, and prostasphere assays

Acinar morphogenesis (AM) assays were carried out using a previously published protocol [52] with modifications [4]. Briefly, 2000 early passage RWPE-1 cell lines (80–85% confluent) were suspended in assay media (KSFM with 5 ng/ml EGF, 2% bovine calf serum and 2% basement membrane extract (Cultrex phenol red-free BME, Bio-Techne Ltd)) and plated in eight-well microslides (Nunc) coated with 40 μl BME per well. CM from control and DKK3-silenced WPMY-1 cells cultured in parallel in assay media for 48 h were collected, centrifuged, and used for AM assays. The remainder was kept at 4 °C for subsequent media changes every 2 days. AM was evaluated as described in other studies [4, 7]. Five photos (×100 magnification) per well were taken at days 4, 6, 7, and 8 using an Axiovert S 100 microscope (Zeiss) and processed with MetaMorph (Molecular Devices) or using an Eclipse TE2000-U microscope (Nikon) and Image-Pro (Media Cybernetics Inc.). The numbers of regular, notched and deformed acini were counted using ImageJ software. Each experiment was repeated in triplicate wells three or four times. Recombinant human (rh) TGFBI (1 μg/ml) and rhECM-1 (100 ng/ml) (R&D Systems) were added to acini every 2 days. Colony formation assays were carried out by plating 200 RWPE-1 shCTRL (NS11) and shDKK3 (sh6) cells in triplicate in 12-well plates for 10 days, changing media every 3 days. Cell colonies were washed with PBS, stained for 20 min using crystal violet (0.2% w/v crystal violet, 20% methanol, 0.4% v/v paraformaldehyde in PBS) and washed again with PBS prior to counting colonies by eye. Prostasphere assays were carried out as previously described [53]. Briefly, shCTRL (NS11) or shDKK-3 (sh6) RWPE-1 cells were plated in quadruplicate in 24-well ultra-low attachment plates (Corning) at 125, 250, or 500 cells per well in DMEM/F-12 with GlutaMAX, antibiotics, B-27 Supplement (Thermo Fisher), 10 ng/ml EGF, and 2 ng/ml bFGF (PeproTech). The SHIP1 inhibitor K118 (Tebu-Bio) was freshly dissolved in water and used at the concentrations indicated. Images of prostaspheres were captured by a camera connected to an Olympus inverted microscope. The total numbers of spheres formed and spheres larger than 125 nm in diameter were evaluated 7 days after plating.

### Gene reporter assays

Gene reporter assays were carried out using pGL3-CAGA12-luc (CAGA-luc), which encodes the luciferase gene fused to 12 repeats of a Smad-binding element [54] and pRL-TK (Promega) as a control. 200,000 cells per well of a 12-well plate were cultured overnight in media without antibiotics and transfected with 100 ng pRL-TK and 400 ng CAGA-luc per well using Lipofectamine LTX with Plus reagent. After 4–5 h, 0.5 ml growth medium was added to each well together with 0.5 ml of medium containing SB431542 (Sigma), SB525334 (Bio-Techne), or an equal volume of vehicle (DMSO). After 24 h, cells were lysed in 200 μl per well passive lysis buffer (Promega), frozen and thawed, centrifuged at 15,000×*g* for 1 min and subjected to luciferase assays using the Dual-Glo® Luciferase assay kit (Promega) or Luciferase Assay Kit (PJK, Germany), according to manufacturers’ instructions, on a Vector luminometer.

### Cell proliferation assays

Cells plated in flasks with 10% FCS DMEM and antibiotics were grown to 80% confluence prior to plating for proliferation assays. Eight thousand cells per well were plated in triplicate in 24-well plates and incubated at 37 °C. Cells were fixed with ice-cold MeOH and stained with 0.2% crystal violet in 20% MeOH at days 1, 3, 5, and 7. Stained cells were solubilized in 10% acetic acid and 100 μl per well was transferred in duplicate to a 96-well plate for measurement of absorbance at 595 nm using an OptiMax plate reader. For co-culture proliferation assays, 50,000 sh30 or sh6 cells in KSFM with 5 ng/ml EGF, 2% bovine calf serum, and 600 μl of 1% BME were plated on 0.4 μm pore inserts in six-well plates. The wells below the inserts were plated with 100,000 WPMY-1 cells in 10% FCS DMEM. Cells were incubated for 48 h at 37 °C and fixed using cold MeOH, stained with 0.2% crystal violet and visualized and counted.

### Invasion and migration assays

PC3 cells starved for 24 h in serum-free RPMI were placed on 8 μm pore Polycarbonate Membrane Transwell inserts in 24-well plates. For invasion assays, inserts were either pre-coated with 1% BME in serum free RPMI and allowed to dry overnight or were carried out using 24-well Corning BioCoat Matrigel Invasion Chambers (SLS Ltd., UK). The assay media contained CM collected after 48 h of serum-free culture of control and DKK3-silenced WPMY-1 cells. PC3 cells were added to the inserts in duplicate in 200 μl serum-free CM, and 800 μl of 10% FCS RPMI was added to the wells below. After 48 h, invaded cells were fixed in cold MeOH, stained with 0.2% crystal violet in 20% MeOH and at least five pictures were taken per insert at ×100 magnification using an Eclipse TE2000-U microscope (Nikon) and Image-Pro (QImaging, Surrey, BC, Canada). Image J software was used to count the numbers of invaded cells. Invasion and migration were also assessed after treatment with 100 nM ARP100 (Santa Cruz Biotechnology). In parallel, PC3 cells were seeded directly in 24-well plates, treated in the same way as the cells used for invasion and migration assays, stained with crystal violet and the numbers used to normalize the migration and invasion data for cell number. To determine the impact of TGFBI and ECM-1 on PC3 cell invasion, 10,000 PC3 cells were plated in triplicate for 24 h and treated with serum-free RPMI alone or with 1 μg/ml rhTGFBI, 100 ng/ml rhECM-1 or both. C4-2B and C4-2BR cell invasion assays were carried out as for PC3 cells, except for the numbers of cells plated (50,000) and the invasion time (24 h).

### Proteome profiler array

Proteome profiling was carried out using the Human Soluble Receptor Array Kit (ARY012, R&D Systems). Prior to harvesting CM, 10^6^ cells were plated in 60 mm dishes in 10% FCS DMEM for 24 h. Five photos per plate were taken using a TE2000-U microscope (Nikon) and cells were counted using Image J. Cells were then washed gently in serum-free DMEM and incubated in serum-free DMEM (~3 ml) with the exact volume adjusted to cell number. After 24 h, CM were collected, centrifuged, and stored at −80 °C. The levels of Dkk-3 in CM were determined by western blotting prior to use and to ensure the absence of intracellular proteins (by blotting for Smad3). Arrays were probed according to the manufacturer’s instructions using 1 ml of CM. Two experiments were carried with independently generated samples of CM. The results were analyzed by densitometry of multiple exposures of the arrays to X-ray film.

### Histochemistry

Tissue arrays (TMAs) with samples from a total of 99 Prostate cancer patients on six arrays were provided by the Imperial Cancer Biomarker Resource Centre (ICBRC) after approval from Imperial CRUK Steering Committee (Tissue Bank application number Project R15043), following patient consent and approval from the local research ethics committee (ref: ICHTB HTA; licence: 12275; REC Wales approval: 12/WA/0196). A total of four sections were provided per patient, two with benign tissue and two with cancer. Nine cores did not contain cancer and were excluded from the analysis. Adjacent or near-adjacent sections of the TMAs were stained for SMA, vimentin, Dkk-3, pan-cytokeratin (CK), TGFBI, and ECM-1. Slides were incubated in a dry oven at 60 °C for 1 h, dewaxed using Histoclear 3 times for 10 min and hydrated by incubation in 100% EtOH twice for 1 min, 70% EtOH for 30 s and immersion in dH_2_0 for 5 min. Antigen retrieval was by immersion in 10 mM Na citrate pH 6.0 in a small glass cup and heating in a microwave oven at 560 W for 8 (Dkk-3), 15 (TGFBI), or 10 (ECM-1) min, and cooling for 30 min. Slides were washed three times in PBS for 5 min on a rocking platform. Endogenous peroxidase was quenched by immersion in 3% H_2_O_2_ for 5 min (ECM-1 for 3 min) and washing three times in PBS for 5 min. Slides were incubated in blocking buffer (Dkk-3 10% horse serum (HS) in PBS, pan-cytokeratin 3% BSA in PBS, TGFBI and ECM-1 5% goat serum in PBS) for 20 min and incubated with primary antibodies (Supplementary Table 1) in blocking buffer overnight at 4 °C. Slides were washed three times in PBS for 5 min, incubated with biotin-conjugated secondary antibodies (1:200, Vector Laboratories) in blocking buffer for 30 min at RT. Bound antibodies were detected using Vectastain Elite ABC Standard kit (Vector Laboratories), according to manufacturer’s instructions. Pictures were taken using a Leica DM750 microscope. Staining intensities were scored independently by two people (ZAS and RMK), any divergences in scores were re-evaluated until consent was found. Scoring for Dkk-3 was repeated by a histopathologist (IZ). Each core was scored based on the staining intensity as 0 (no staining), 1 (weak staining), 2 (moderate staining), or 3 (strong staining, if present).

### Statistical analysis

Results are presented as the mean ± standard deviation (SD). All experiments were repeated at least three times. Statistical evaluations were performed with GraphPad Prism 5.0 (GraphPad, La Jolla, CA, USA) using two-sided Student’s *t* test for single comparisons or one-way analysis of variance (ANOVA) with post hoc Tukey for multiple group comparisons. A two-tailed *p* value < 0.05 was considered to indicate statistical significance. For TMA analysis, patients were divided into low (0, 1) and high (2, 3) expression and Gleason scores ≥ 4 + 3 and ≤ 3 + 4 and analyzed by one-way v2 test, Chi-squared test with Yates correction or Fisher’s exact test, two-sided, available on the VassarStats website (http://vassarstats.net/). Correlation analysis was calculated using Phi-correlations. Analyses were performed using SPSS v16 (IBM Corp., Somers, NY, USA) or MATLAB-MathWorks software. For multiple comparisons in acinar morphogenesis assays, two-way ANOVA and Tukey’s multiple comparison was done using Graphpad Prism v6.

## Electronic supplementary material

Supplementary Figure legends

Supplementary Tables 1, 2 and 3

Supplementary Figure 1

Supplementary Figure 2

Supplementary Figure 3

Supplementary Figure 4

Supplementary Figure 5

Supplementary Figure 6

Supplementary Figure 7

Supplementary Figure 8

Supplementary Figure 9

Supplementary Figure 10

Supplementary Figure 11

Supplementary Figure 12
