# Supplementary material for: Protective effect of stromal Dickkopf-3 in prostate cancer: opposing roles for TGFBI and ECM-1
Source: Oncogene. 2018 Jun 1;37(39):5305–24. doi: 10.1038/s41388-018-0294-0 (PMC6160402; doi:10.1038/s41388-018-0294-0)
Supplement: Supplementary file 1 — Supplementary Figure legends [file 41388_2018_294_MOESM1_ESM.docx]

**Supplementary Table 1.** Primary antibodies used for western blotting (w) and immunohistochemistry (i)

**Supplementary Table 2.** List of primers used in this study with sequences and concentrations

**Supplementary Table 3.** Clinical and pathological characteristics of the prostate cancer patients in the TMAs.

**Supplementary Figure 1. Scoring system for Dkk-3** (**a**) Examples of Dkk-3 immunohistochemistry in prostate epithelial/cancer cells scored using the quickscore method as 0 (negative), 1 (weak), 2 (moderate), 3 (strong). (**b**) Examples of Dkk-3 immunohistochemistry in stromal cells scored using the quickscore method; staining of epithelial cells in adjacent sections using anti-pan cytokeratin (CK) and stromal cells using SMA are also shown.; scale bars 112 µm.

**Supplementary Figure 2. Characterization of DKK3-silenced cells** (**a**) Western blots of extracts from shCTRL (PSM3) and shDKK3 (Wsh8) WPMY-1 cells were probed for Dkk-3 and GAPDH. Bar chart shows densitometry analysis of Dkk-3 normalized to GAPDH in extracts; error bars represent SD, n=3, *p<0.001 by Student’s t test. (**b**) Proliferation assays for 8000 parental (WPMY-1), shCTRL (PSM2, NPSM) and shDKK3 (Wsh7 and Wsh8) WPMY-1 cells plated in triplicate in 24-well plates and analyzed by Crystal violet staining; n =3.

**Supplementary Figure 3. Effects of DKK3 silencing on Smad2 and Smad4 in WPMY-1 cells** (**a**) Western blots of extracts from WPMY-1 and shDKK3 (Wsh8) cells cultured for 24 h with (+) or without (-) 10 ng/ml TGF-β1, in serum-free medium were probed for Smad2 and GAPDH as a loading control. (**b**) Densitometry analysis of Smad2 normalized to GAPDH, comparing control cells (WPMY-1 and shCTRL (PSM2)) and shDKK3 (Wsh7, Wsh8) WPMY-1 cells in untreated cells (-) or cell treated with TGF-β1(+), n=3. (**c**) As for (a) probing for Smad4. (**d**) As for (b) for Smad4, n=3.

**Supplementary Figure 4. Effects of DKK3 silencing on MMP2 and SMA in WPMY-1 cells** (**a**) Western blots of extracts from WPMY-1 and shDKK3 (Wsh8) WPMY-1 cells cultured for 24 h with (+) or without (-) 10 ng/ml TGF-β1, in serum-free medium were probed for MMP2 and GAPDH as a loading control. (**b**) Densitometry analysis of MMP2 normalized to GAPDH, comparing control cells (WPMY-1 and shCTRL PSM2) and shDKK3 (Wsh7, Wsh8) WPMY-1 cells in untreated cells (-) or cell treated with TGF-β1(+), n=3. (**c**) As for (a) probing for SMA. (**d**) As for (b) for SMA, n=4.

**Supplementary Figure 5. (a** and **b) FACS plots of RWPE-1 cells transfected with GFP reporter plasmids** Examples of FACS plots for shCTRL (NS11) and shDKK3 (sh6) RWPE-1 cells transfected with s-SHIP promoter-driven GFP (**a**) and a CMV promoter-driven GFP (**b**); the GFP-positive populations (P5) are in green. (**c**) Titration of effects of K118 (24 h treatment) on viability of shCTRL and shDKK3 RWPE-1 cells; graph shows representative experiment carried out in triplicate, n=2.

**Supplementary Figure 6. WPMY-1 cell CM promotes normal acinar morphogenesis in a Dkk-3-dependent manner** (**a**) shDKK3 (sh6) RWPE-1 cells were cultured with assay medium (control) or CM from shCTRL (NPSM) or shDKK3 (Wsh7) WPMY-1 cells and normal (regular), notched and deformed acini were counted at day 7; error bars show SD, n=3, *p < 0.05. (**b**) shDKK3 (sh30) RWPE-1 cells were cultured with assay medium (control) or CM from shCTRL (NPSM) or shDKK3 (Wsh7) WPMY-1 cells and acini counted as in (a); error bars show SD, n=4, *, **, ***, **** p < 0.05, p < 0.01, p < 0.001, p < 0.0001, respectively. (**c**) AM assays (day 7) using shCTRL (NS14) RWPE-1 cells cultured with assay media (control) or CM from shCTRL (NPSM) or shDKK3 (Wsh7) WPMY-1 cells; error bars show SD, n=4, *, **, p < 0.05, p < 0.01, respectively.

**Supplementary Figure 7. Dkk-3-silenced WPMY-1 cell CM increases PC3 cell invasion** (**a**) Cell invasion assays using PC3 cells cultured with CM from shCTRL (NPSM) and shDKK3 (Wsh7) WPMY-1 cells, n=3, * p<0.05 by Student’s t test. Left, representative photos of invaded cells stained with crystal violet. (**b**) Cell invasion assays in the presence of serum-free medium (Ctrl) and CM from WPMY-1 cells, n=3.

**Supplementary Figure 8. Antibody array analysis of CM from control and DKK3-silenced cells** Arrays were incubated with CM from (**a**) shCTRL (PSM2) and shDKK3 (Wsh8) WPMY-1 cells or (**c**) shCTRL (NS11) and shDKK3 (sh6) RWPE-1 cells; two experiments were carried out using two independent batches of CM. The circles show spots for TGFBI (red) and ECM-1 (green). (**b** and **d**) Densitometry analysis of the signals for TGFBI and ECM-1, normalized to the averaged signal from the reference control spots; error bars show SD, n=2.

**Supplementary Figure 9. Scoring system for TGFBI (a)** Examples of TGFBI immunohistochemistry in epithelial/cancer cells scored using the quickscore method as 0 (negative), 1 (weak), 2 (moderate), 3 (strong); staining of epithelial cells in adjacent sections using anti-pan cytokeratin (CK) is also shown. (**b**) Examples of TGFBI immunohistochemistry in stromal cells scored using the quickscore method; staining of stromal cells (SMA) in near adjacent sections is also shown; scale bars 112 µm.

**Supplementary Figure 10. Scoring system for ECM-1 (a)** Examples of ECM-1 immunohistochemistry in epithelial/cancer cells scored using the quickscore method as 0 (negative), 1 (weak), 2 (moderate), 3 (strong); staining of epithelial cells in adjacent sections using anti-pan cytokeratin (CK) is also shown. (**b**) Examples of ECM-1 immunohistochemistry in stromal cells scored using the quickscore method; staining of stromal cells (SMA) in near adjacent sections is also shown; scale bars 112 µm.

**Supplementary Figure 11**. Gene expression and relapse-free survival of PCa patients Kaplan-Meier curves for DKK3 (GSE70768) (**a**), ECM-1 (GSE7076) (**b**) and TGFBI (GSE70768) (**c**); HR hazard ratio, UCI upper confidence interval, LCI lower confidence interval, graphs from PROGgeneV2.

**Supplementary Figure 12.** (**a** and **b**) q-RT-PCR analysis of the indicated genes showing average relative expression in shCTRL (PSM3) (**a**) and shDKK3 (Wsh8) (**b**) WPMY-1 cells either untreated or treated with 1 ug/ml TGFBI for 24 h; n=3, * p<0.05 by Student’s t test. (**c** and **d**) q-RT-PCR analysis of TGFBI showing average relative expression in shCTRL (PSM3) (**c**) and shDKK3 (Wsh8) (**d**) WPMY-1 cells either untreated or treated with 10 ng/ml TGF-β1, 1 μM SB431542 or 1 μg/ml TGFBI for 24 h; n=3, * p<0.05 by Student’s t test. (**e**) Average Ct values for TGFBI and ECM1 in the indicated cell lines; n > 2. (**f**) q-RT-PCR analysis of the indicated genes in C4-2B and C4-2B MDVR cells, relative to C4-2B cells; n=2.
