# Supplementary material for: Protective effect of stromal Dickkopf-3 in prostate cancer: opposing roles for TGFBI and ECM-1
Source: Oncogene. 2018 Jun 1;37(39):5305–24. doi: 10.1038/s41388-018-0294-0 (PMC6160402; doi:10.1038/s41388-018-0294-0)
Supplement: Supplementary file 2 — Supplementary Tables 1, 2 and 3 [file 41388_2018_294_MOESM2_ESM.docx]

| **SUPPLEMENTARY TABLE 1** | | |  |
| --- | --- | --- | --- |
| Antigen | Source | Species | Dilution |
| Dkk-3 | R&D AF1118 | goat | 1:200 (i) |
|  |  |  | 1:500 (w) |
| ECM-1 | Proteintech 11521-1-AP | rabbit | 1:500 (w) |
|  |  |  | 1:200 (i) |
|  | Santa Cruz sc-365946 | mouse | 1:500 (w) |
| TGFBI (b-IgH3) | Santa Cruz sc-28660 | rabbit | 1:50 (i) |
|  |  |  | 1:50 (w) |
| Pan cytokeratin | Thermo Scientific MA5-13156 (AE1/AE3) | mouse | 1:200 (i) |
| Smooth muscle actin (SMA) | Abcam [4A4] ab119952 | mouse | 1:50 (i) |
|  |  |  | 1:1000 (w) |
| MMP2 | Invitrogen | mouse | 1:400 (i) |
|  |  |  | 1:1000 (w) |
| Smad2 | Cell Signaling sampler kit #9963 | rabbit | 1:1000 (w) |
| Smad3 | Abcam ab40854 | Rabbit | 1:2000 (w) |
| Smad4 | Cell Signaling sampler kit #9963 | rabbit | 1:1000 (w) |
| phospho-Smad3 | R&D AB3226 | mouse | 1:1000 (w) |
| GAPDH | Santa Cruz sc-59541 | mouse | 1:5000 (w) |

| **SUPPLEMENTARY TABLE 2** | |  |  |
| --- | --- | --- | --- |
| **Gene** | **Forward Primer (5’ → 3’)** | **Reverse Primer (5’ → 3’)** | **Ratio (nM)** |
| *DKK3* | TCATCACCTGGGAGCTAGAG | TTCATACTCATCGGGGACCT | 500/500 |
| *TGFBI* | CACCAAGAGAACGGAGCAGA | GCCTCCGCTAACCAGGATTT | 300/300 |
| *VEGFA* | CCTTGCTGCTCTACCTCCAC | ATGATTCTGCCCTCCTCCTT | 900/900 |
| *ECM1* | ATTTGGCTGTTGCTTCTGCT | TCTTGAAAGTGCTCTGGCCT | 600/600 |
| *SOX2* | GCACATGAACGGCTGGAGCAACG | TGCTGCGAGTAGGACATGCTGTAGG | 900/900 |
| *Oct-04* | GACAACAATGAAAATCTTCAGGAG | CTGGCGCCGGTTACAGAACCA | 900/900 |
| *NANOG* | CAGCTGTGTGTACTCAATGATAGATTT | ACACCATTGCTATTCTTCGGCCAGTTG | 300/900 |
| *ALDH1A1* | TGTTAGCTGATGCCGACTTG | TTCTTAGCCCGCTCAACACT | 900/900 |
| *S-SHIP* | CTTGATGTTCACCTTGTCCCCTGC | TCAACGTCGACTTTGAGCTGC | 300/300 |
| *ACTG2* | TACCCCATTGAACACGGCAT | TGCTCTTCAGGTGCTACA CG | 600/600 |
| *ANGPT1* | TGGGGGAGGTTGGACTGTAA | GCCAATATTCACCGGAGGGA | 300/900 |
| *36B4* | GTGTTCGACAATGGCAGCAT | AGACACTGGCAACATTGCGGA | 500/500 |

| **SUPPLEMENTARY TABLE 3** |  |
| --- | --- |
| **Characteristic** | **Number of patients** |
| Total number of patients | 99 |
| Patient sections with tumour | 89 |
| Age range (years) | 32-99 |
| Median age | 63 |
| Average age | 62 |
| Low-moderate grade prostate cancer (Gleason ≤ 3+4) | 77 |
| High grade prostate cancer (Gleason ≥ 4+3) | 22 |
| Acute or chronic inflammation | 27 |
| Perineural invasion | 42 |
| Vascular invasion | 2 |
| Lymphovascular invasion | 4 |
| Lymph node metastasis | 1 |

| **SUPPLEMENTARY FIGURE 11A** | | |  |
| --- | --- | --- | --- |
| **H.R** | **LCI 95%** | **UCI 95%** | **p value** |
| 0.47 | 0.22 | 1 | ***0.0492*** |
| Category | HIGH | LOW |  |
| Samples | 55 | 55 |  |
| No of events | 6 | 12 |  |
| Median survival | NA | NA |  |
| Low conf int (95%) | 1710 | 1650 |  |
| Upp conf int | NA | NA |  |
| **SUPPLEMENTARY FIGURE 11B** | | |  |
| **H.R** | **LCI 95%** | **UCI 95%** | **p value** |
| 0.46 | 0.23 | 0.94 | ***0.0341*** |
| Category | HIGH | LOW |  |
| Samples | 46 | 45 |  |
| No of events | 20 | 25 |  |
| Median survival | 2949 | 1275 |  |
| Low conf int (95%) | 1847 | 712 |  |
| Upp conf int | NA | NA |  |
| **SUPPLEMENTARY FIGURE 11B** | | |  |
| **H.R** | **LCI 95%** | **UCI 95%** | **p value** |
| High | 55 | 10 | NA |
| Category | HIGH | LOW |  |
| Samples | 55 | 55 |  |
| No of events | 10 | 8 |  |
| Median survival | NA | NA |  |
| Low conf int (95%) | 1710 | 1650 |  |
| Upp conf int | NA | NA |  |
| **SUPPLEMENTARY FIGURE 12E** | | |  |
| **Cell line** | **TGFBI** | **ECM1** |  |
| C4-2B | 31.5 | 30.5 |  |
| PC3 | 26 | 32 |  |
| RWPE-1 | 18 | 23 |  |
| WPMY-1 | 15 | 24.5 |  |
