# Supplementary figures and images for: Protective effect of stromal Dickkopf-3 in prostate cancer: opposing roles for TGFBI and ECM-1
Source: Oncogene. 2018 Jun 1;37(39):5305–24. doi: 10.1038/s41388-018-0294-0 (PMC6160402; doi:10.1038/s41388-018-0294-0)

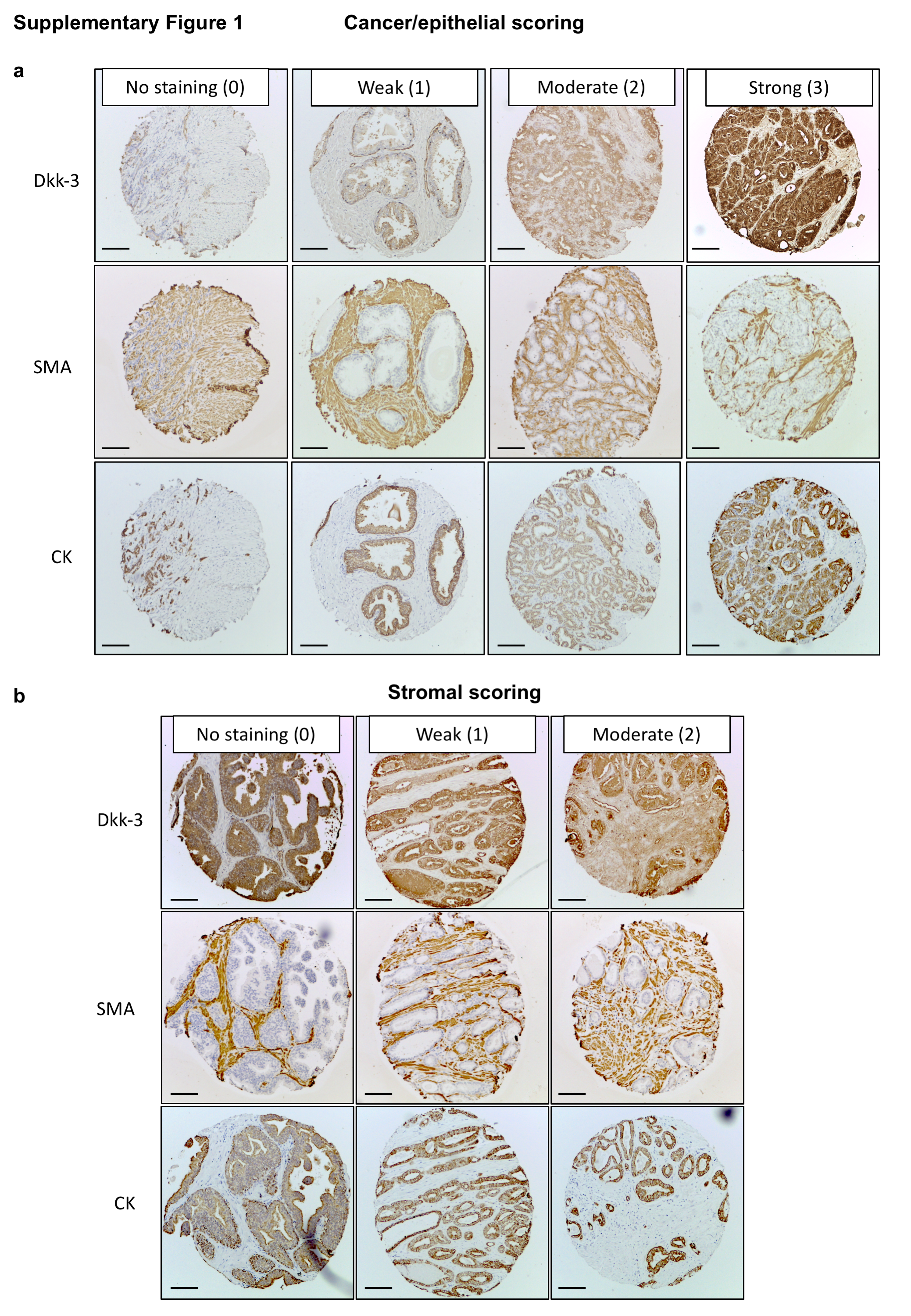

Supplement: Supplementary file 3 — Supplementary Figure 1 [file 41388_2018_294_MOESM3_ESM.tif]

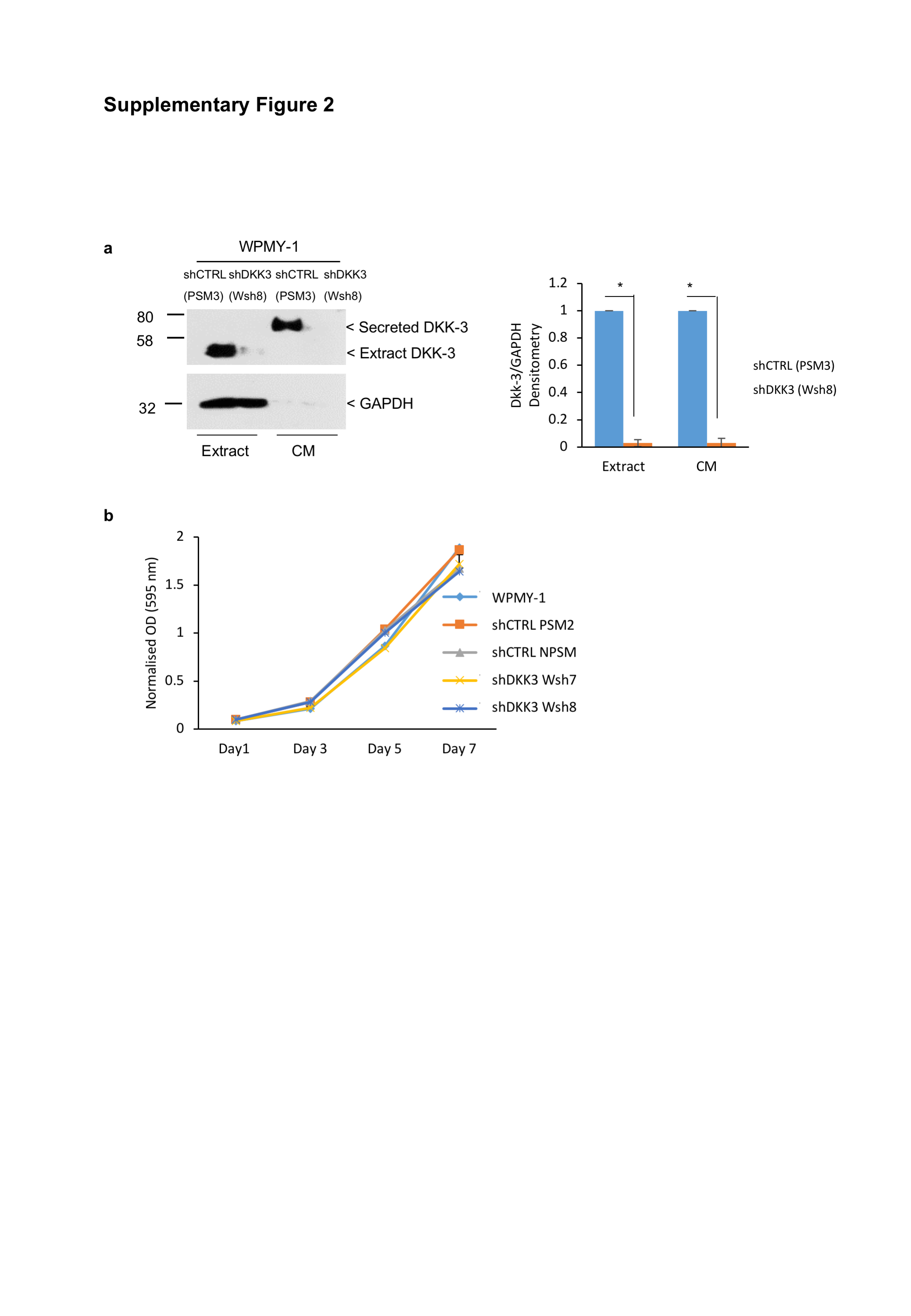

Supplement: Supplementary file 4 — Supplementary Figure 2 [file 41388_2018_294_MOESM4_ESM.tif]

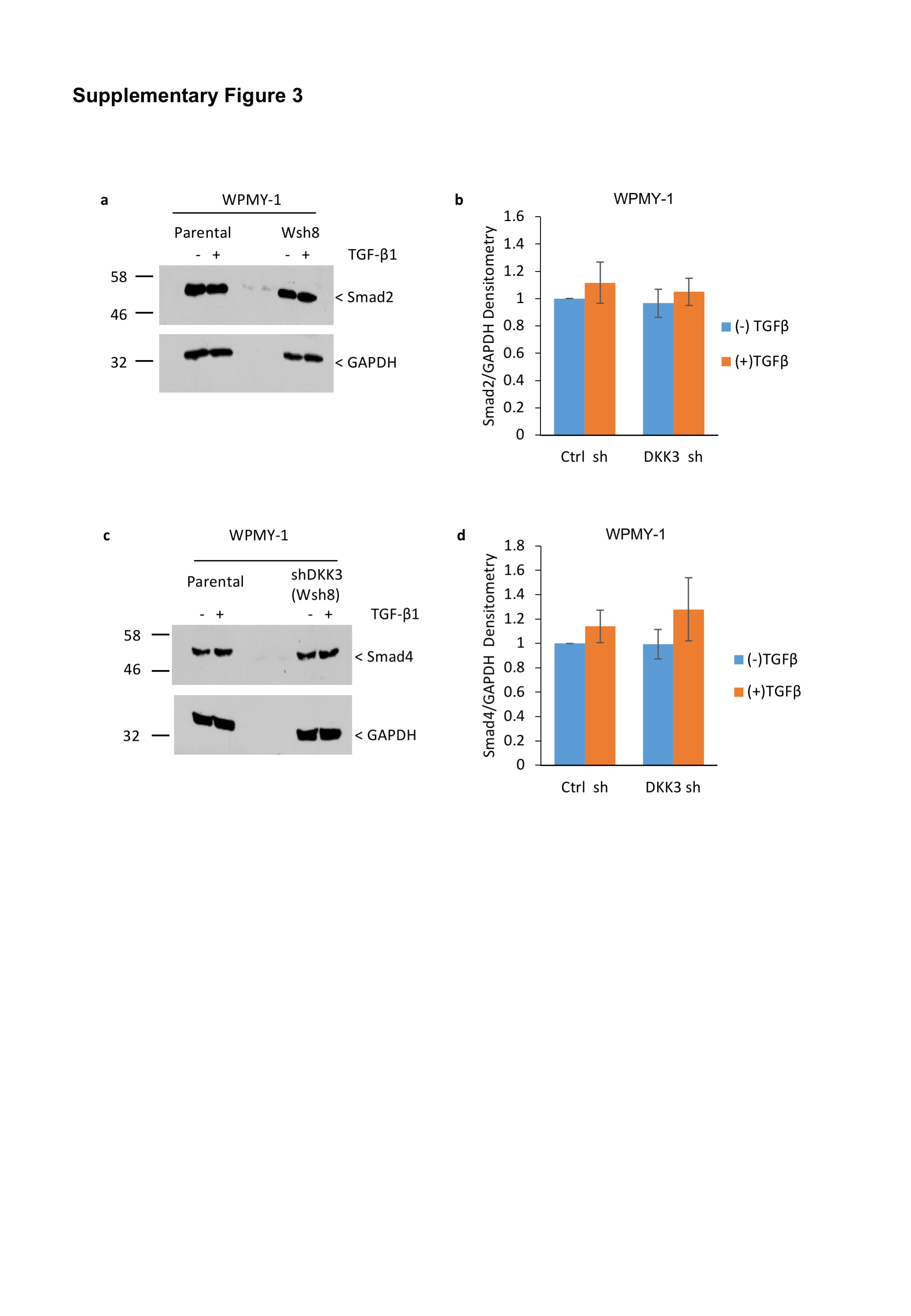

Supplement: Supplementary file 5 — Supplementary Figure 3 [file 41388_2018_294_MOESM5_ESM.tif]

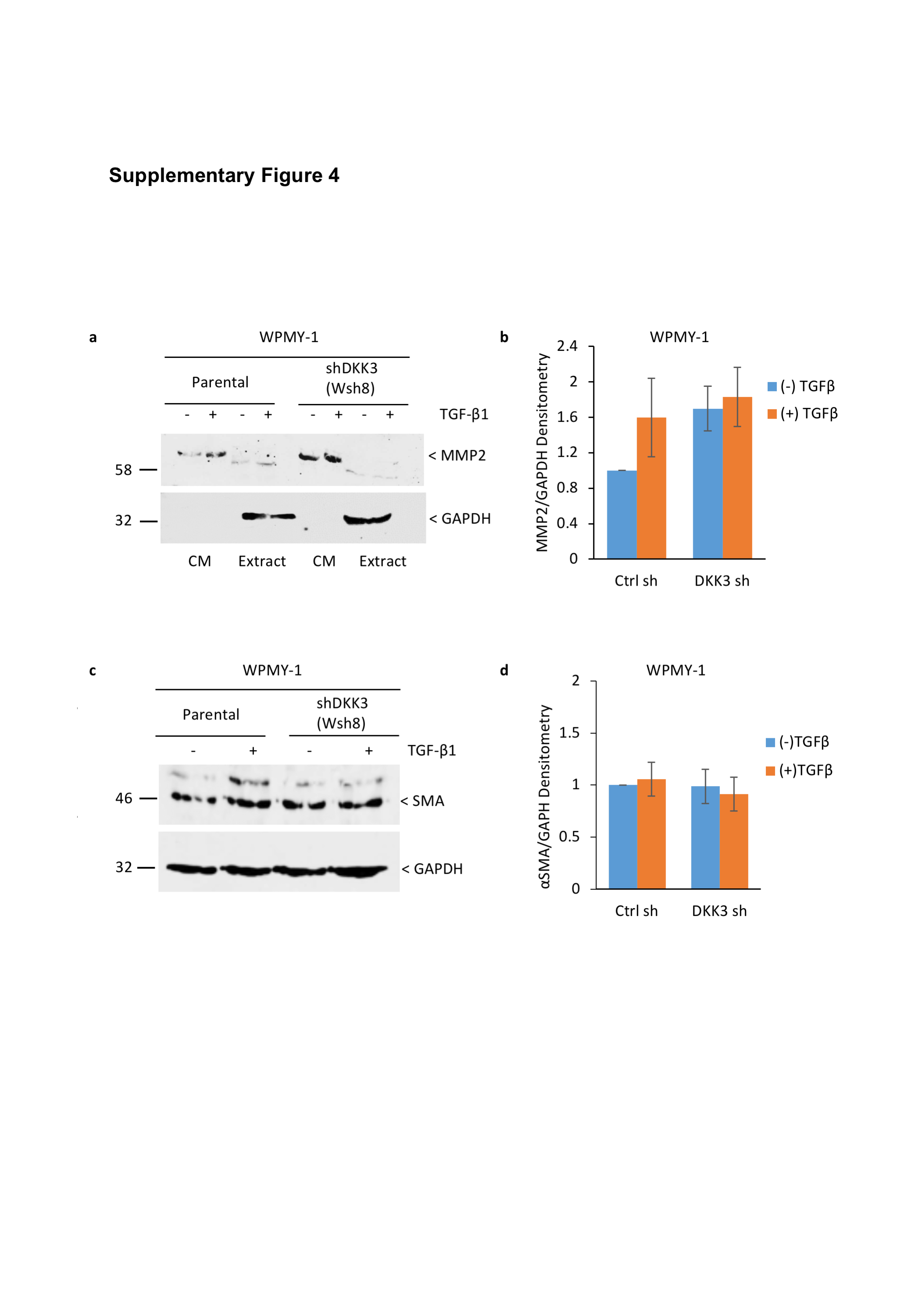

Supplement: Supplementary file 6 — Supplementary Figure 4 [file 41388_2018_294_MOESM6_ESM.tif]

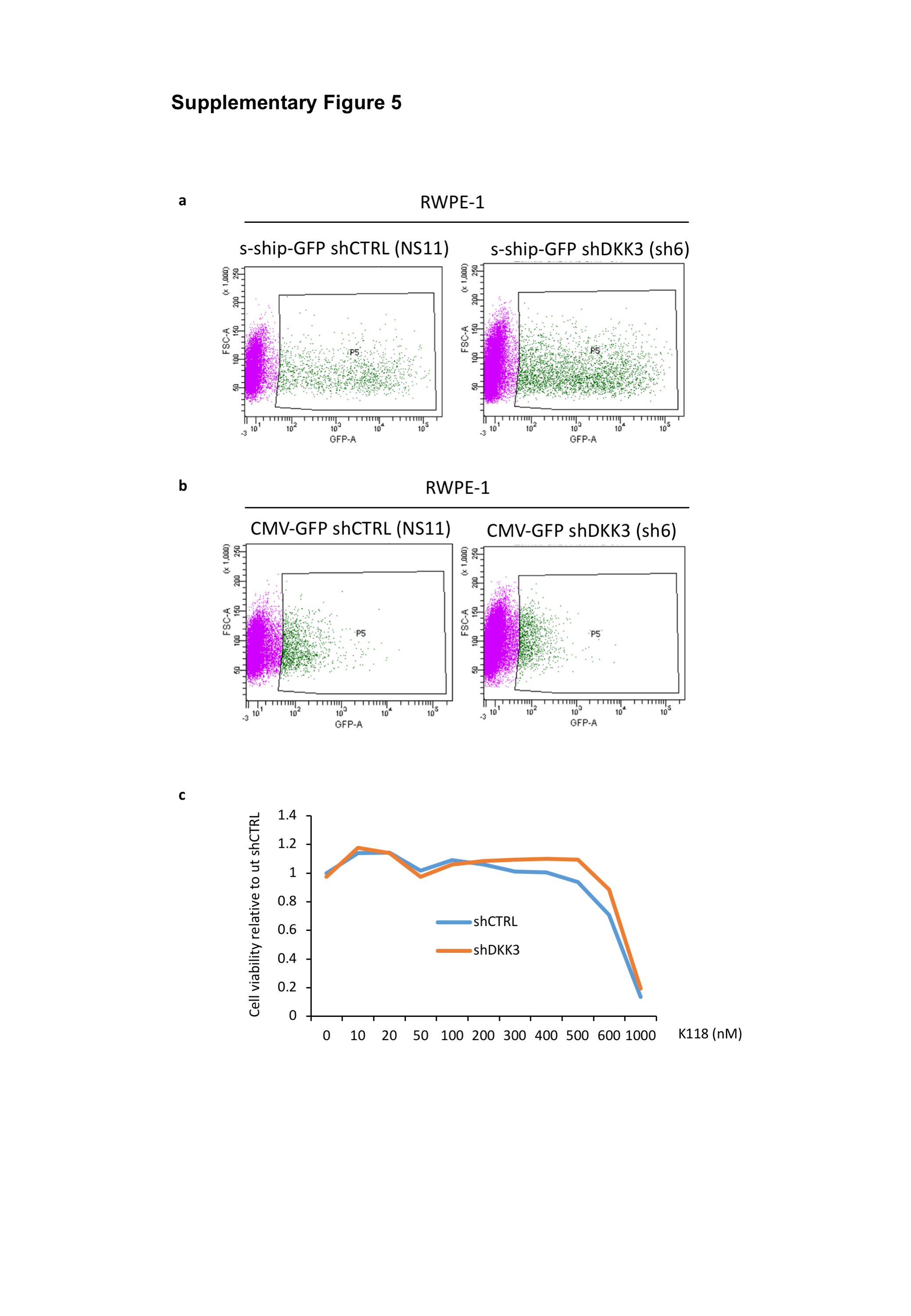

Supplement: Supplementary file 7 — Supplementary Figure 5 [file 41388_2018_294_MOESM7_ESM.tif]

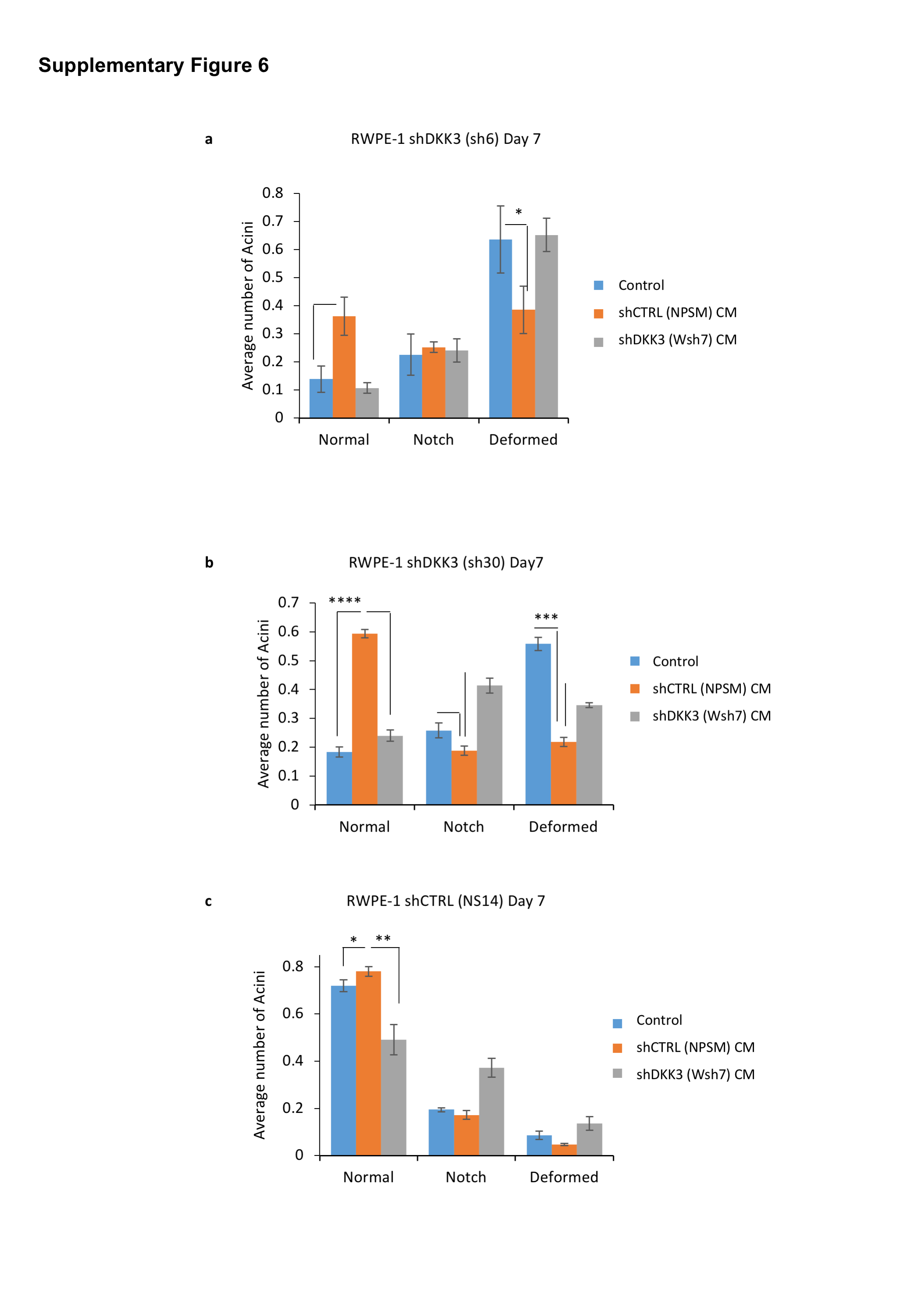

Supplement: Supplementary file 8 — Supplementary Figure 6 [file 41388_2018_294_MOESM8_ESM.tif]

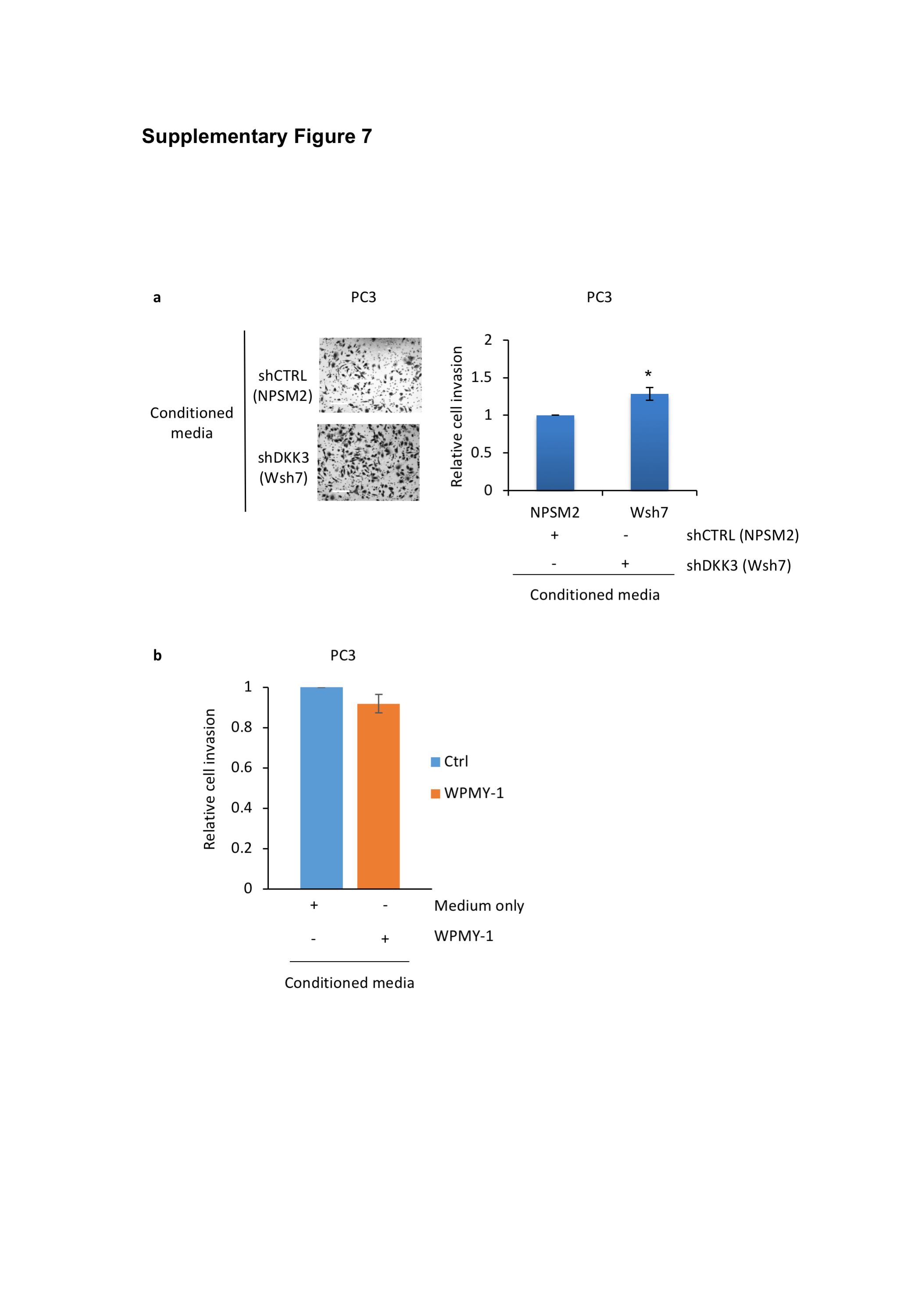

Supplement: Supplementary file 9 — Supplementary Figure 7 [file 41388_2018_294_MOESM9_ESM.tif]

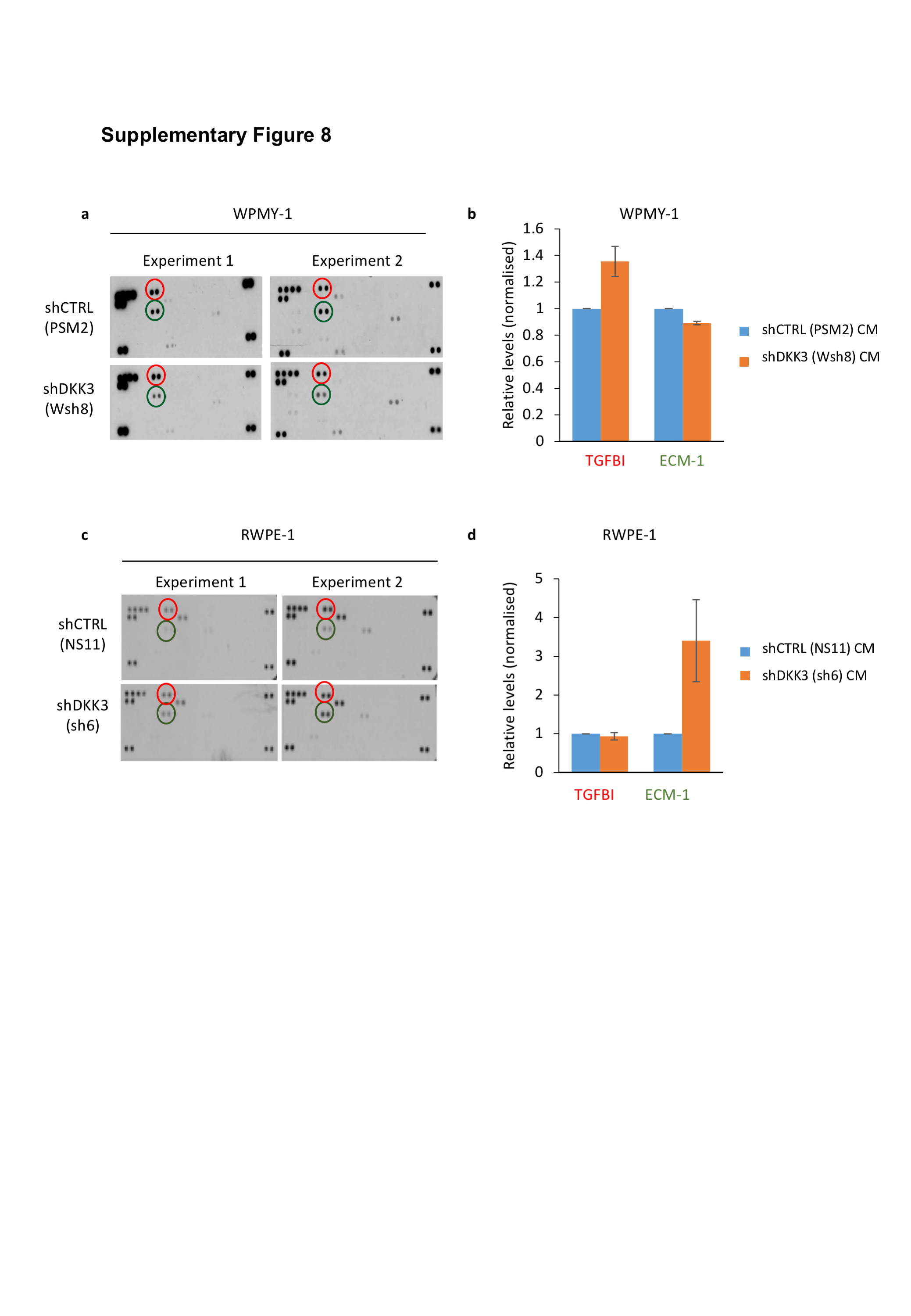

Supplement: Supplementary file 10 — Supplementary Figure 8 [file 41388_2018_294_MOESM10_ESM.tif]

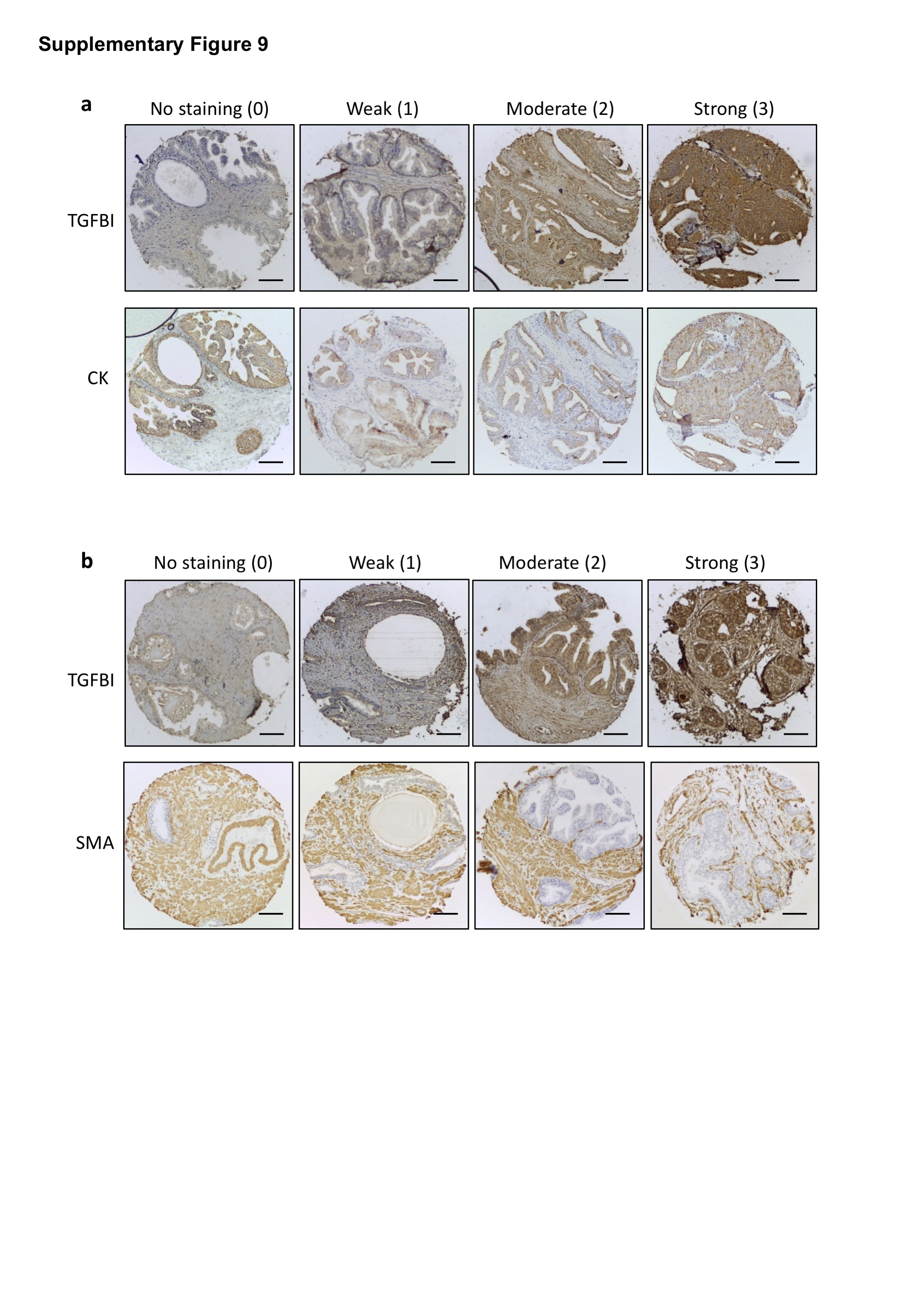

Supplement: Supplementary file 11 — Supplementary Figure 9 [file 41388_2018_294_MOESM11_ESM.tif]

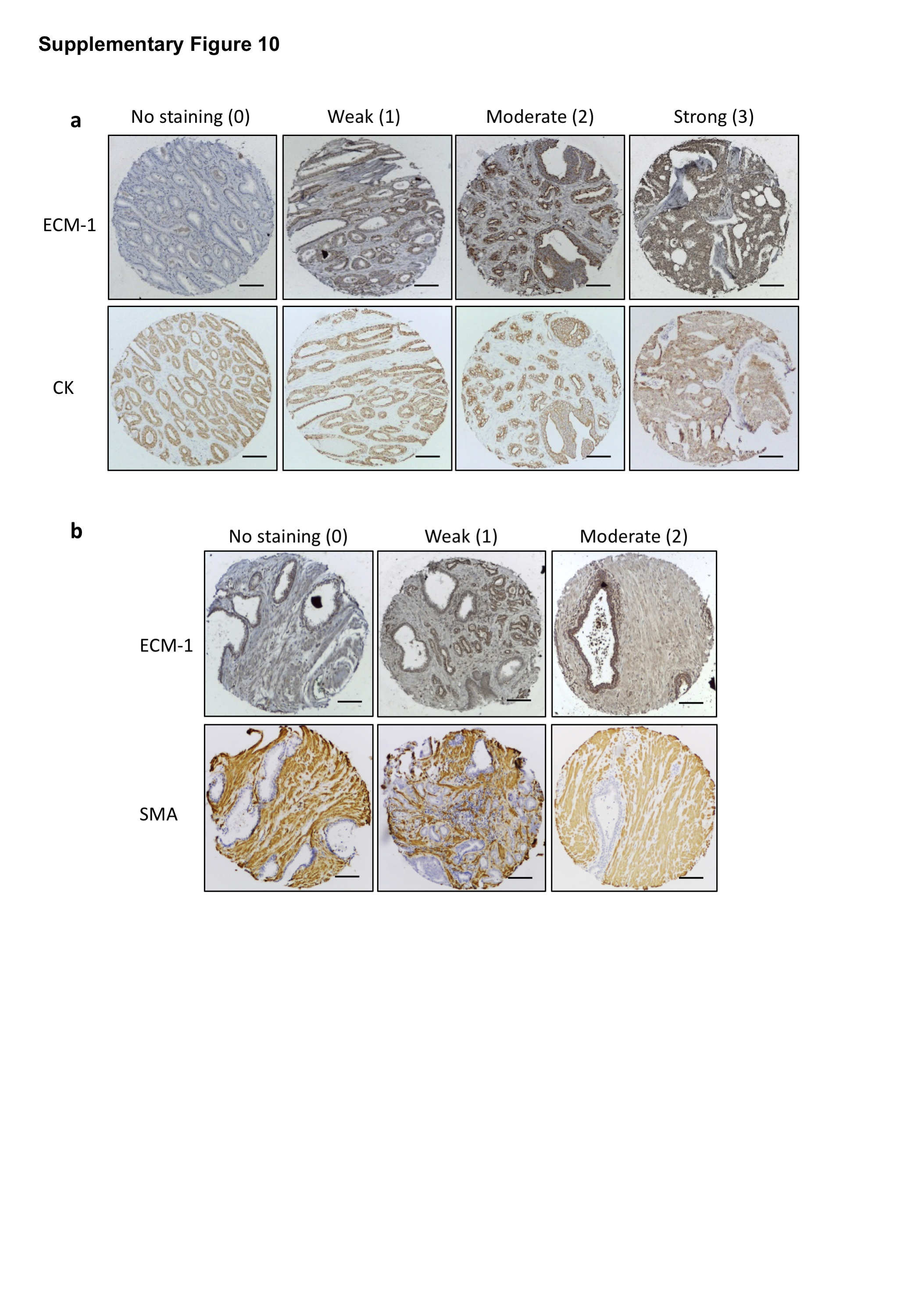

Supplement: Supplementary file 12 — Supplementary Figure 10 [file 41388_2018_294_MOESM12_ESM.tif]

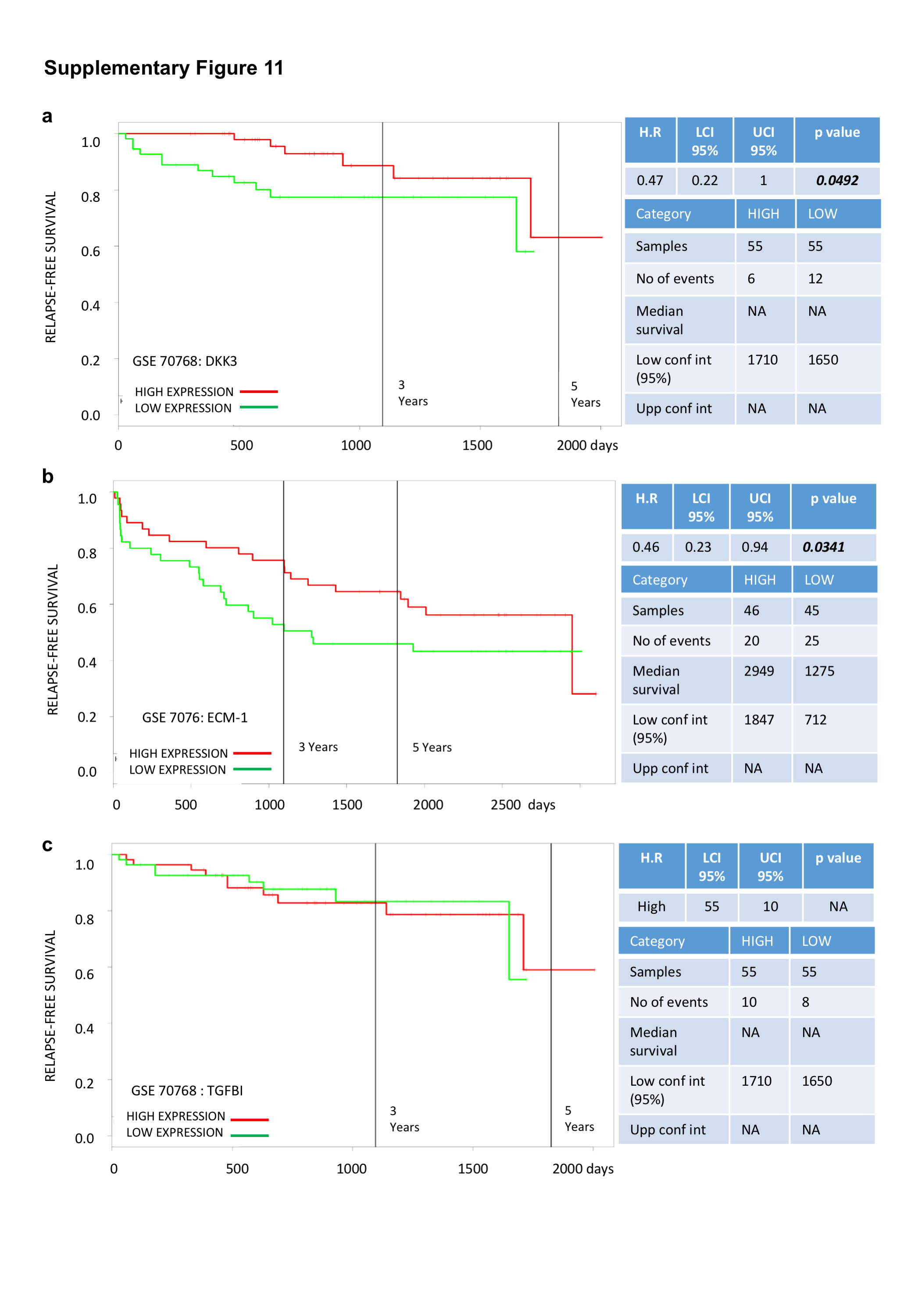

Supplement: Supplementary file 13 — Supplementary Figure 11 [file 41388_2018_294_MOESM13_ESM.tif]

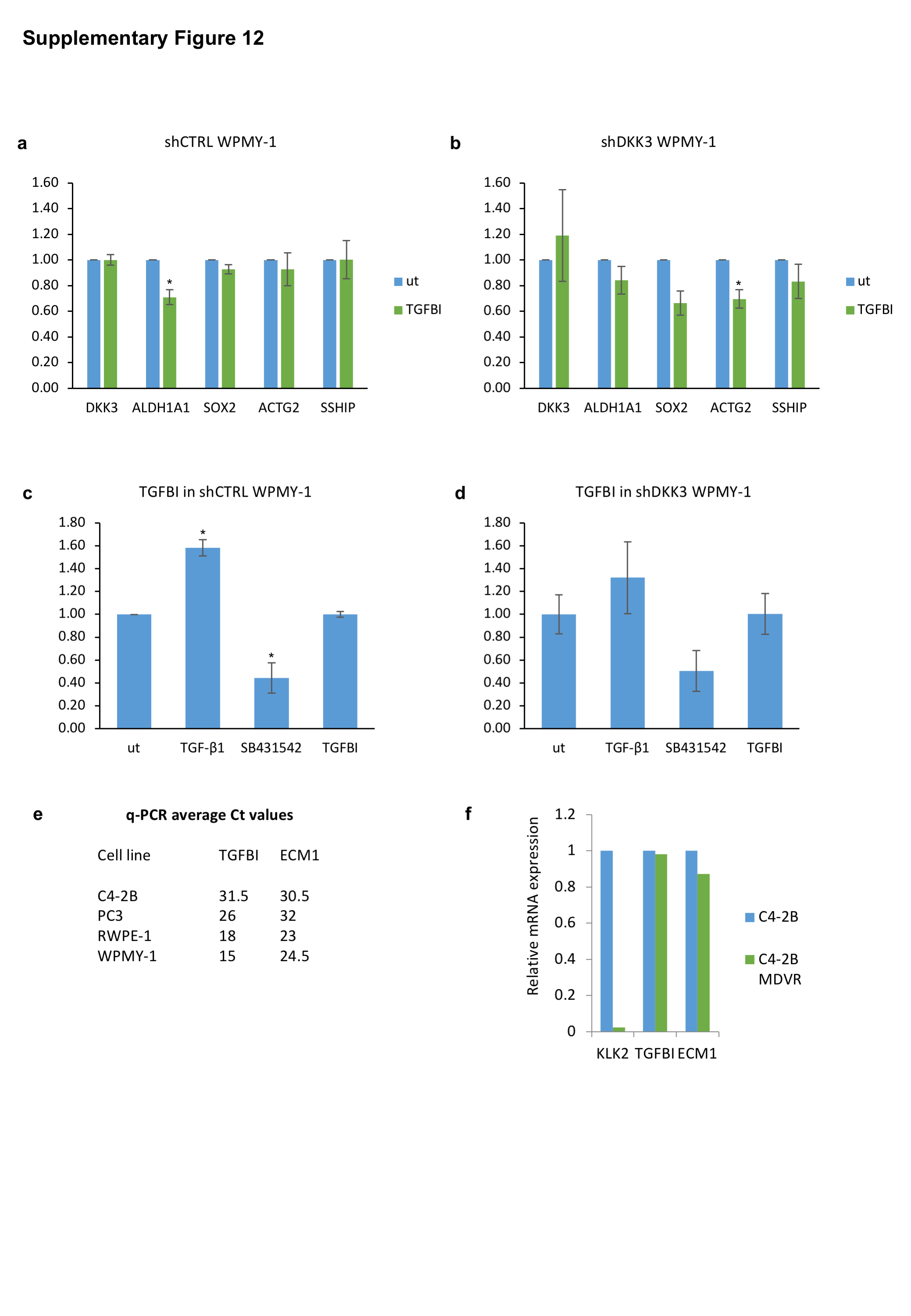

Supplement: Supplementary file 14 — Supplementary Figure 12 [file 41388_2018_294_MOESM14_ESM.tif]
